# Impact of treatment methods on recycled concrete aggregate performance: a comprehensive review

**DOI:** 10.1007/s11356-025-36497-y

**Published:** 2025-05-31

**Authors:** Dulshi Peiris, Chamila Gunasekara, David W. Law, Yulin Patrisia, Vivian W. Y. Tam, Sujeeva Setunge

**Affiliations:** 1 Civil and Infrastructure Engineering, RMIT University, Melbourne, Australia; 2 Teaching and Learning Department, Universitas Palangka Raya, Palangka Raya, Indonesia; 3 School of Engineering, Design and Built Environment , Western Sydney University, Sydney, Australia

**Keywords:** Construction and demolition waste, Recycled aggregate concrete, Treatment methods, Circular economy, Sustainable materials, Recycled concrete aggregate

## Abstract

The disposal of construction and demolition waste (CDW) poses a critical global environmental challenge, driven by the low recyclability of concrete due to the limitations of recycled aggregates (RA). These aggregates suffer from the high porosity of attached mortar, resulting in elevated water absorption, reduced density, and diminished mechanical performance, restricting their application in structural concrete. This comprehensive review examines innovative treatment methodologies—either removing or strengthening the attached mortar layer—aimed at mitigating these issues. The paper synthesizes findings from over 150 studies, offering a critical analysis of treatment effects on physical, mechanical, and durability properties of RAs and their corresponding concretes. The review highlights the most effective removal treatment methods, including ball milling and autogenous cleaning, and strengthening methods such as carbonation, polymer impregnation, and nano-silica treatments, demonstrating significant improvements in density, strength, and chloride resistance. Recommendations are provided for scaling these treatments, integrating durability testing, and exploring life-cycle assessment to ensure the environmental and economic feasibility of RA applications. This work underscores the transformative potential of RA treatments in advancing circular economy principles, making recycled concrete a sustainable solution for structural and non-structural applications.

## Introduction

Natural coarse aggregates (or simply “natural aggregates” or NAs) are currently being utilized in large quantities in the construction industry, depleting natural resources and creating negative impacts on the environment (Pu et al. 2021). Aggregates are integral to the production of concrete, which is the primary construction material, and whose use is increasing due to the continued growth of population and infrastructure. Thus, the identification of alternative sustainable materials is critical to reduce the environmental burden on the use of natural aggregates.

Owing to the rapid urbanization throughout the world, enormous quantities of construction and demolition waste (CDW) is being generated, posing serious environmental challenges. In a global context, CDW constitutes 30–40% of solid municipal waste (Pu et al. 2021). Currently CDW exceeds 12.7 billion tons per year and is predicted to reach 27 billion tons by 2050 (Silva et al. 2024). However, approximately 35% of CDW is still dumped into landfills, contributing to the prevailing environmental crises (Liang et al. 2020).

**Fig. 1:**
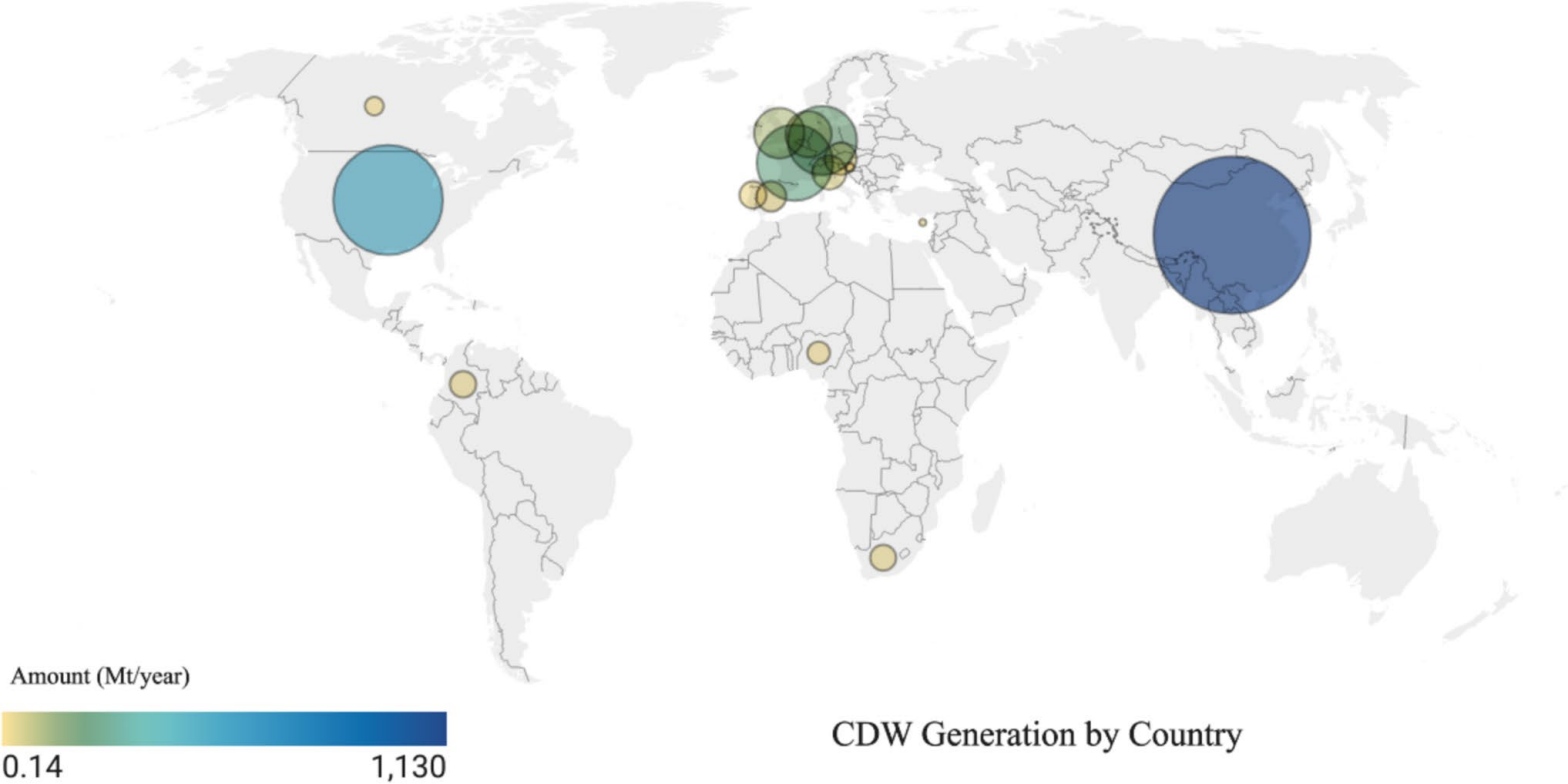
CDW Generation by Country (Soto-Paz et al. 2023)

CDW consists of concrete, bricks, ceramic, glass, plastic, wood, and other building materials. The composition of CDW varies depending on their region of origin (Soto-Paz et al. 2023) and parent sources (Associates 1998). Figure 1 provides a graphical representation of the amount of CDW produced by a selection of countries throughout the world. It can be observed that there is a significant variation in CDW production. China generates the highest amount of CDW at 1130 Mt/year, which is more than twice that of the United States (534 Mt/year) and significantly higher than any European country. Among European countries, Cyprus (0.14 Mt/year) and Croatia (0.68 Mt/year) generate minimal amounts compared to France (246 Mt/year) and Germany (200 Mt/year). The USA, China, Germany, and France contribute the most to global CDW, reflecting their large-scale infrastructure and construction activities. In contrast, developing nations like Nigeria (15 Mt/year) and South Africa (21 Mt/year) have much lower outputs.

**Fig. 2:**
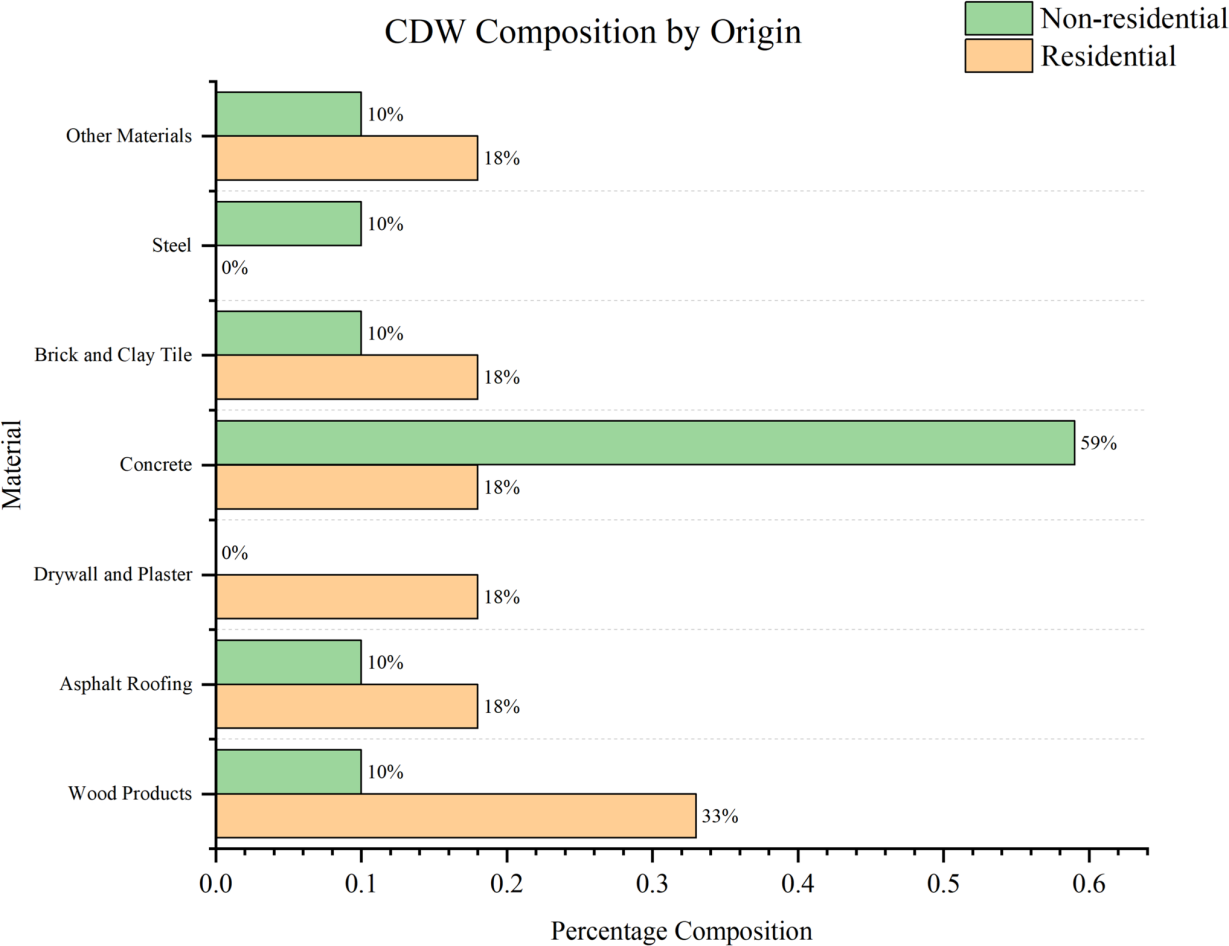
CDW composition by origin (Associates 1998)

**Fig. 3:**
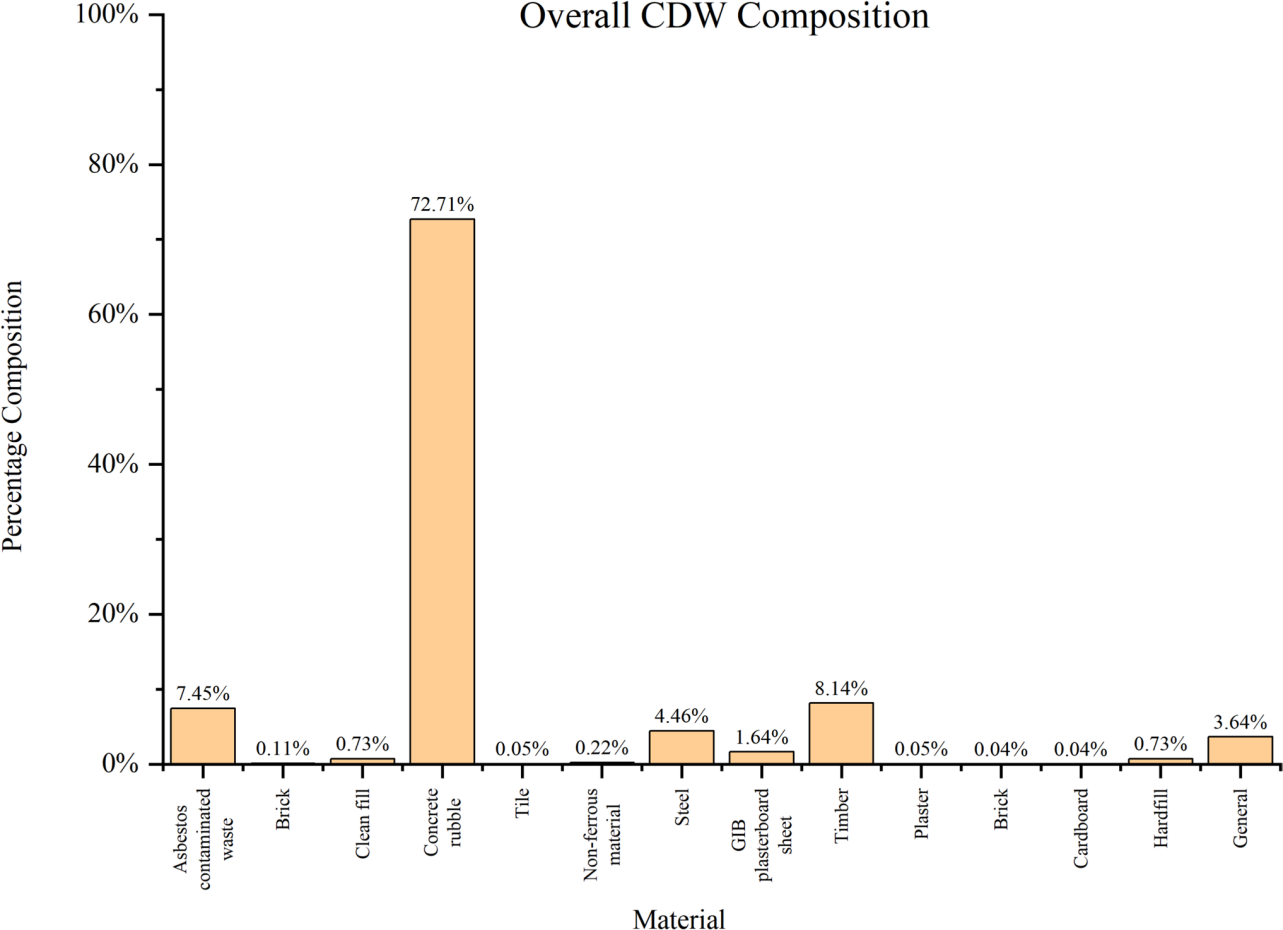
Overall CDW Waste Composition (Shooshtarian et al. 2020)

Figure 2 depicts significant differences between non-residential and residential CDW. Concrete dominates non-residential CDW, accounting for 59%, compared to only 18% in residential waste, reflecting the type of structural materials used in commercial and industrial buildings. Conversely, wood products (33%) and drywall/plaster (18%) are more prevalent in residential CDW, likely due to their common use in housing construction. Brick and clay tiles, asphalt roofing, and other materials also show have higher amounts in residential waste. Steel, while present in non-residential waste (10%), is absent in residential waste. These variations indicate that waste management strategies should be tailored based on building type, with a focus on concrete recycling in commercial demolition. Concrete is the most utilized construction material in the world, more than thirty billion tonnes being manufactured globally per year (Monteiro et al. 2017). The universal popularity of concrete due to its intrinsic properties such as strength, durability, customizability of strength and shape, and ease of maintenance (Neville and Brooks 2010). Therefore, when taken as a whole throughout the construction industry (Fig. 3), over 70% of CDW is reportedly comprised of concrete (Akhtar and Sarmah 2018; Shooshtarian et al. 2020).

**Fig. 4:**
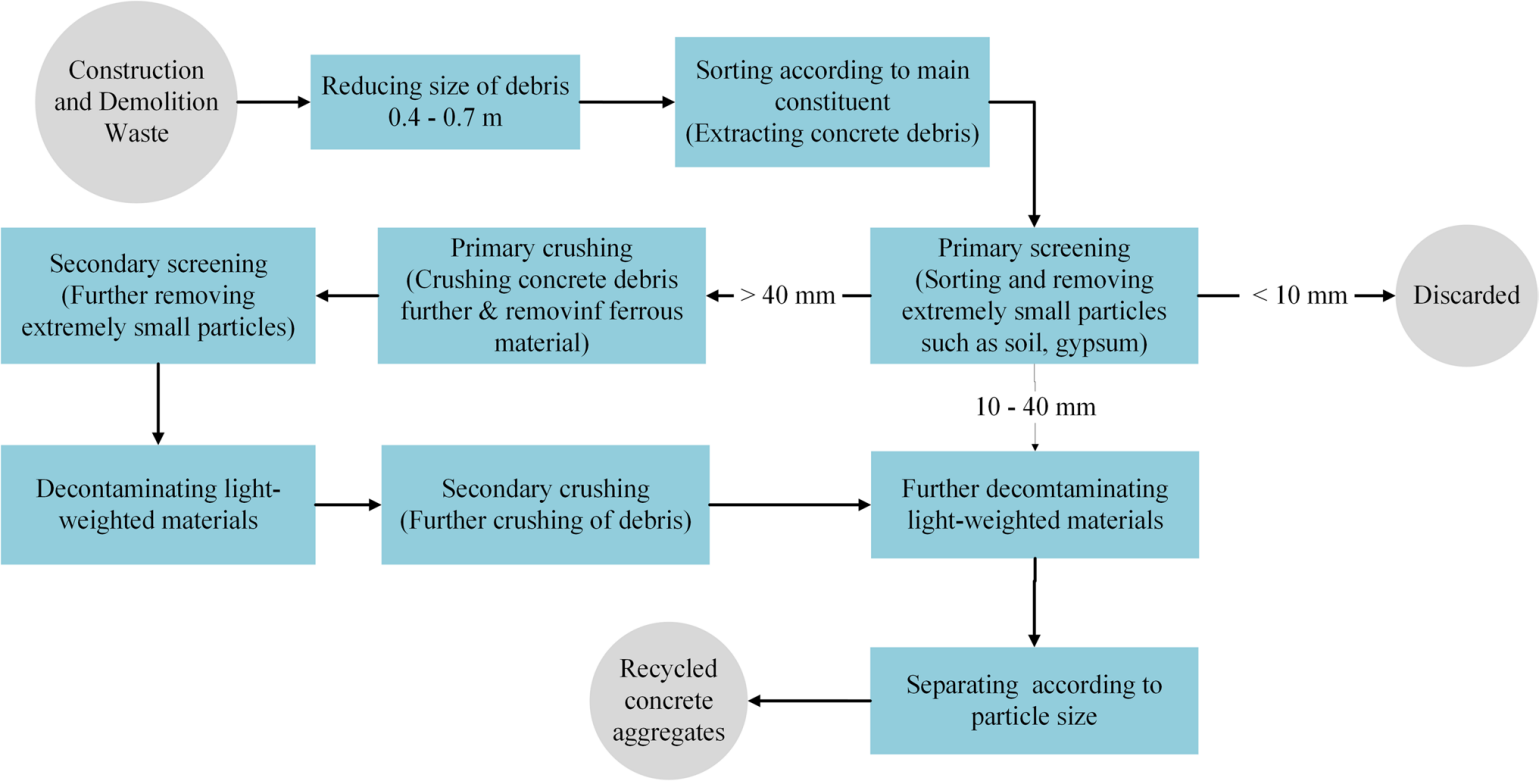
Flowchart of the recycling process of CDW as described by Wang et al. (2021)

Recycling of concrete has emerged as an effective way for managing CDW, contributing to the creation of a circular economy. The process of generating recycled aggregates from waste concrete has continuously evolved, with the latest advancement being a 9-step mechanism by Wang et al. (2021), as depicted in Fig. 4. First, large pieces of debris are broken down into the range 0.4–0.7 m using hydraulic crushers and pneumatic hammers. They are then sorted according to their main constituents such as concrete, wood, steel, glass, plastic, etc. Materials that contain very small particles (such as soil and gypsum) are screened and removed. Next, the concrete debris is further crushed (primary crushing), and any remaining ferrous material removed. This is followed by a secondary screening to dispose of the remaining soil and other small particles. The debris is then decontaminated by the removal of lightweight materials such as plastic, paper and wood pieces via washing or air-sifting. The debris is crushed once again to further reduce its size and thereby obtain the requisite aggregates. The aggregates are then sanitized a final time to dispose of any residual lightweight material. Finally, the resulting recycled aggregates are divided into two categories according to their particle size: recycled fine aggregates and recycled coarse aggregates, with coarse aggregates comprising more than 50% (Chen et al. 2024).

Despite the extensive recycling process, a layer of mortar attached to the surface of aggregate prevails in RA produced. This mortar is highly porous in nature and possesses several unfavorable properties in the RA, such as rough and irregular surfaces, high water absorption, low density, and high crushing value (Pu et al. 2021). The increased surface roughness and irregularity of RAs give rise to a lower workability in concrete prepared incorporating RAs (also known as “recycled aggregate concrete,” “recycled concrete” or RC), than concrete prepared with natural coarse aggregates (also known as “natural aggregate concrete,” “natural concrete” or NC) (Kurda et al. 2017). To compensate, additional water has to be added to RC. Additionally, because of the higher affinity of RA for water absorption, the water content needs to be increased. This will in turn result in concrete with higher porosity and lower compressive strength (Amhudo et al. 2018). The lower density of RA can be viewed as advantageous for the production of lightweight concrete. However, this lower density is as a result of the porosity of the attached mortar layer which gives rise to other inherently inferior properties making it ultimately unfavorable. The aggregate crushing value (ACV) is a determining factor for the resistance of aggregates to failure under a compressive load. The lower the ACV, the better the resistance to crushing (Pacana et al. 2021). RAs generally possess a higher ACV than NAs contributing to weaker concrete (Das et al. 2021).

Compressive strength is one of the most important mechanical properties of concrete. Building standards impose specific compressive strength requirements for a concrete element to be classified as a structural element. The Australian standard AS 3600 proposes at least 25 MPa for 28-day compressive strength while EN 1992-1-1:2004 (Eurocode 2) sets a limit of 20 MPa, based on standard cylindrical samples. Unfortunately, the compressive strength of RC is known to be significantly diminished when compared with NC. Consequently, as splitting tensile strength, flexural tensile strength and the elastic modulus limitations are derived from the compressive strength they are also likely to be inferior in RC, rendering them unsuitable in a structural context (Xuan et al. 2016). Long-term properties such as water absorption, rate of carbonation, resistance to chloride and sulphate diffusion, creep and shrinkage contribute to the longevity of a concrete structure. Eurocode suggests a maximum water absorption of 5% and a maximum water-cement ratio of 0.45–0.50 for exposure classes XC1–XC4 to prevent carbonation. AS 3600 does not provide an exact limit on water absorption; however, a maximum water-cement ratio of 0.50 is recommended. Additionally, a minimum cover of 30 mm is imposed to resist carbonation. A maximum 0.2% Cl⁻ by mass of cement for exposure classes XS1–XS3 and a maximum 0.8 kg/m^3^ Cl⁻ for marine exposures are stipulated by Eurocode 2 and AS 3600 respectively. To minimize sulphate ingress, both the Eurocode and AS 3600 suggest utilizing a sulphate resisting cement with low C_3_A content. The creep and shrinkage impositions by Eurocode are 1.1–2.5 and 200–600 μξ while those of AS 3600 are 2.0–3.0 and 400–800 μξ. It has also been established that generally these durability properties are unsatisfactory with regards to RC (Yehia et al. 2015). Therefore, the application of RC is generally limited to only non-structural concrete elements. At present, several countries permit the use of recycled aggregates in structural components, though some remain cautious. As research advances, it is anticipated that recycled aggregates will be adopted more widely in additional countries. Most countries have set limits on the maximum compressive strength grade or RA replacement ratio, alongside specific standards for density and water absorption, such as Japan (JIS A 5021, 5022, 5023), European nations (RILEM TC 121-DRG), Germany (DIN4226–100), China (GB/T 25177), Australia (AS 2758.1), and Korea (KSF 2527) (Table 1).

**Table 1:** Standards on recycled aggregates in different countries

Country	Specification	Limitations on RA replacement (de Andrade Salgado and de Andrade Silva 2022)	Limitations on RA properties (de Andrade Salgado and de Andrade Silva 2022)
United Kingdom	BS 8500–2	20% for C 20/25 and C 40/50 grades	≤ 1% SO4^2−^ contaminants
		100% for C 16/20 grade	Not defined
Australia	AS 2758.1–2014	Not defined	≤ 30% ACV
Brazil	NBR 15116	20% for structural grades	≤ 7% water absorption
Portugal	LNEC E471	25% for C 35/45 grade	≥ 2200 kg/m^3^ dry density ≤ 7% water absorption
		20% for C 40/50 grade	≥ 2200 kg/m^3^ dry density ≤ 7% water absorption
Japan	JIS A 5021	Not defined	≥ 2500 kg/m^3^ dry density ≤ 3% water absorption
Italy	NTC-2008	30% for C 30/37 grade	Not defined
China	DG/TJ 07/008	Not defined	< 10% water absorption < 1% SO4^2−^ contaminants

**Fig. 5:**
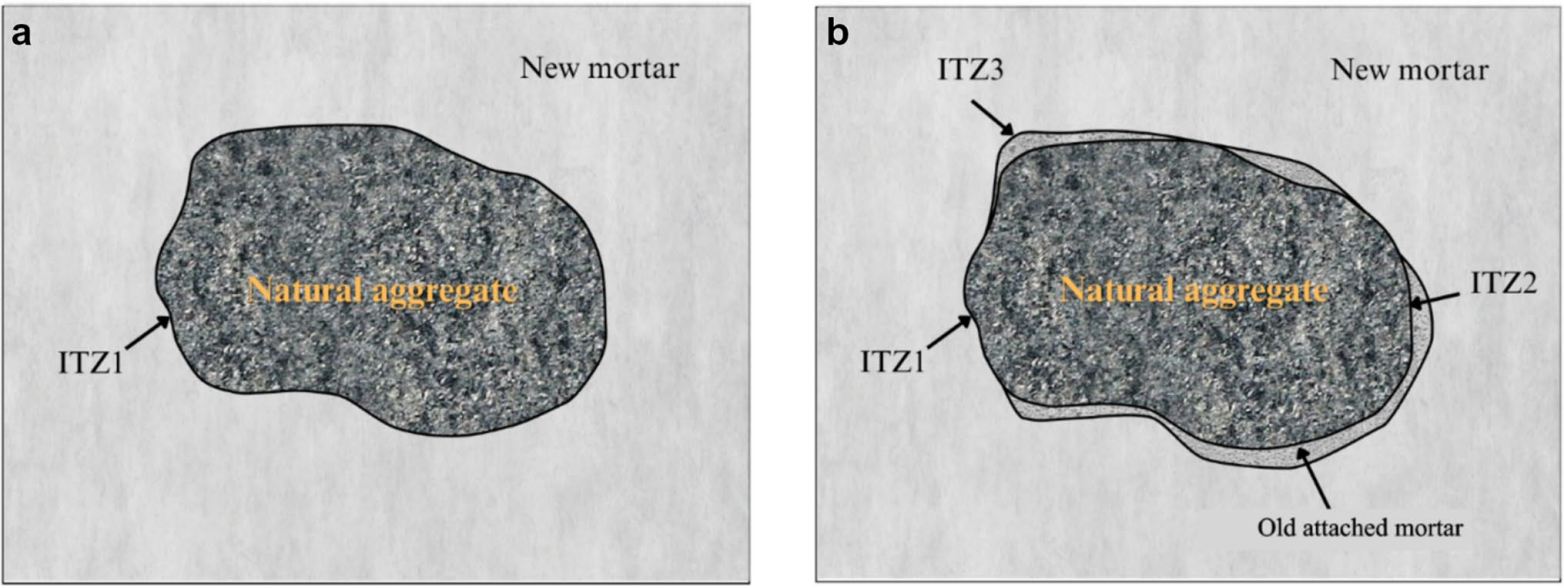
ITZs of (**a**) natural and (**b**) recycled aggregate concrete

Due to the presence of the attached mortar layer, RC possesses three interfacial transition zones (ITZ) as opposed to only one in NC. As shown in Fig. 5, the ITZ between the natural coarse aggregate particle and new mortar (ITZ1) exists in both NC and RC. However, RC comprises of two additional ITZs: one between attached mortar and natural coarse aggregate (ITZ2), and the other between the attached mortar and new mortar (ITZ3). The ITZ3 between attached and new mortar layers in RA has been shown to be weak and gives rise to aforementioned inferior mechanical and durability properties (Xuan et al. 2016). Therefore, many past studies have applied a range of treatments to RA, such as removing the attached mortar layer, strengthening the attached mortar layer or both, to enhance their properties and the performance of RA, in an effort to replace NA in structural elements.

**Fig. 6:**
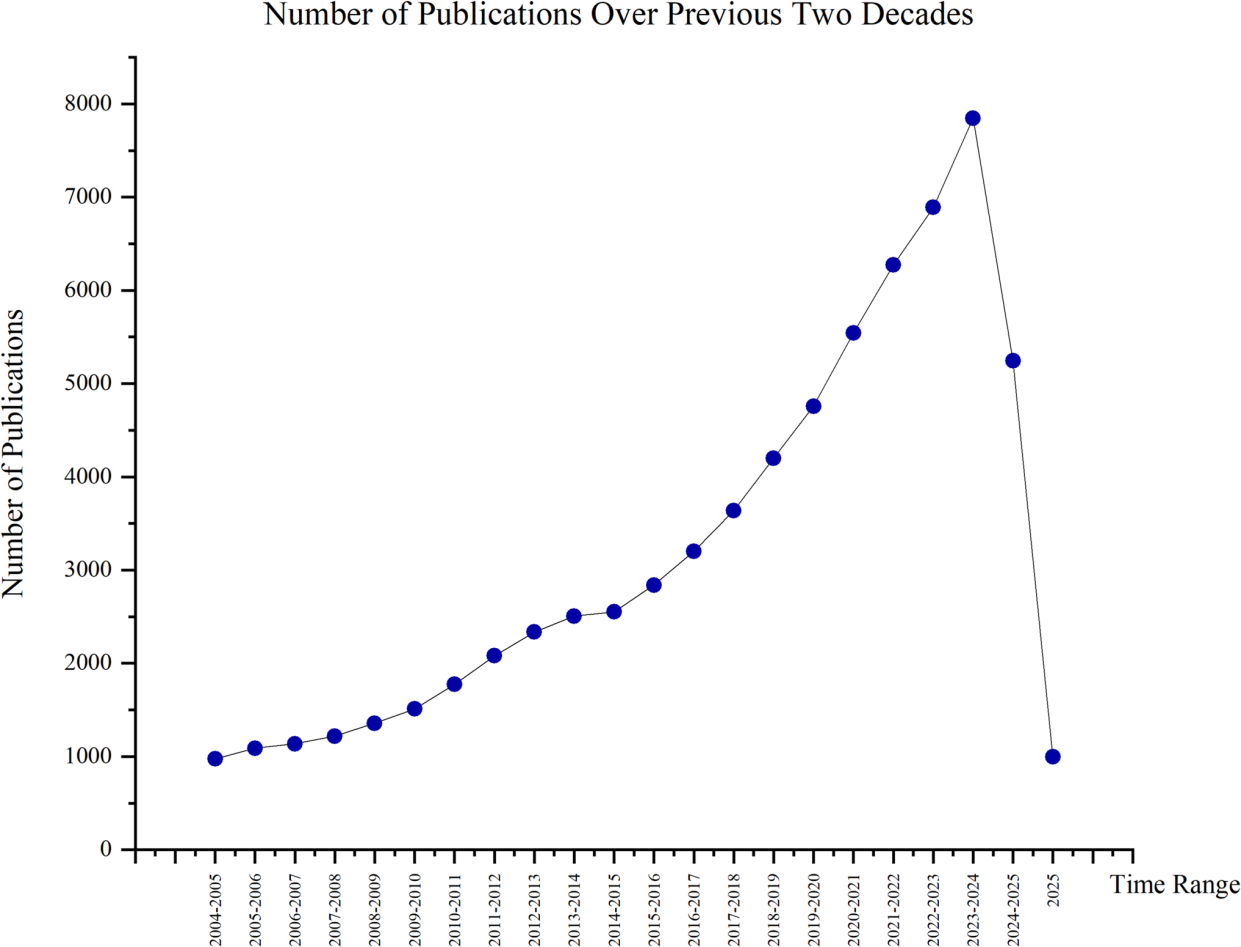
Number of publications over previous two decades

The bibliometric analysis on the treatment of recycled aggregate research from 2004 to 2025 reveals significant global contributions, as shown in Fig. 6. A total of over 50,000 publications have been recorded. The yearly publication trend (January 2004–March 2025) on recycled aggregate treatment shows a steady rise in research interest, with publications increasing from 975 in 2004–2005 to a peak of 7847 in 2023–2024. This growth reflects the expanding global focus on sustainable construction. A notable surge is observed after 2015, with yearly publications exceeding 3000 and continuing to grow. The drop from 2024 onwards is due to incomplete data collection for 2024. This trend underscores the increasing recognition of recycled aggregate treatment as a crucial solution for sustainable construction practices.

**Fig. 7:**
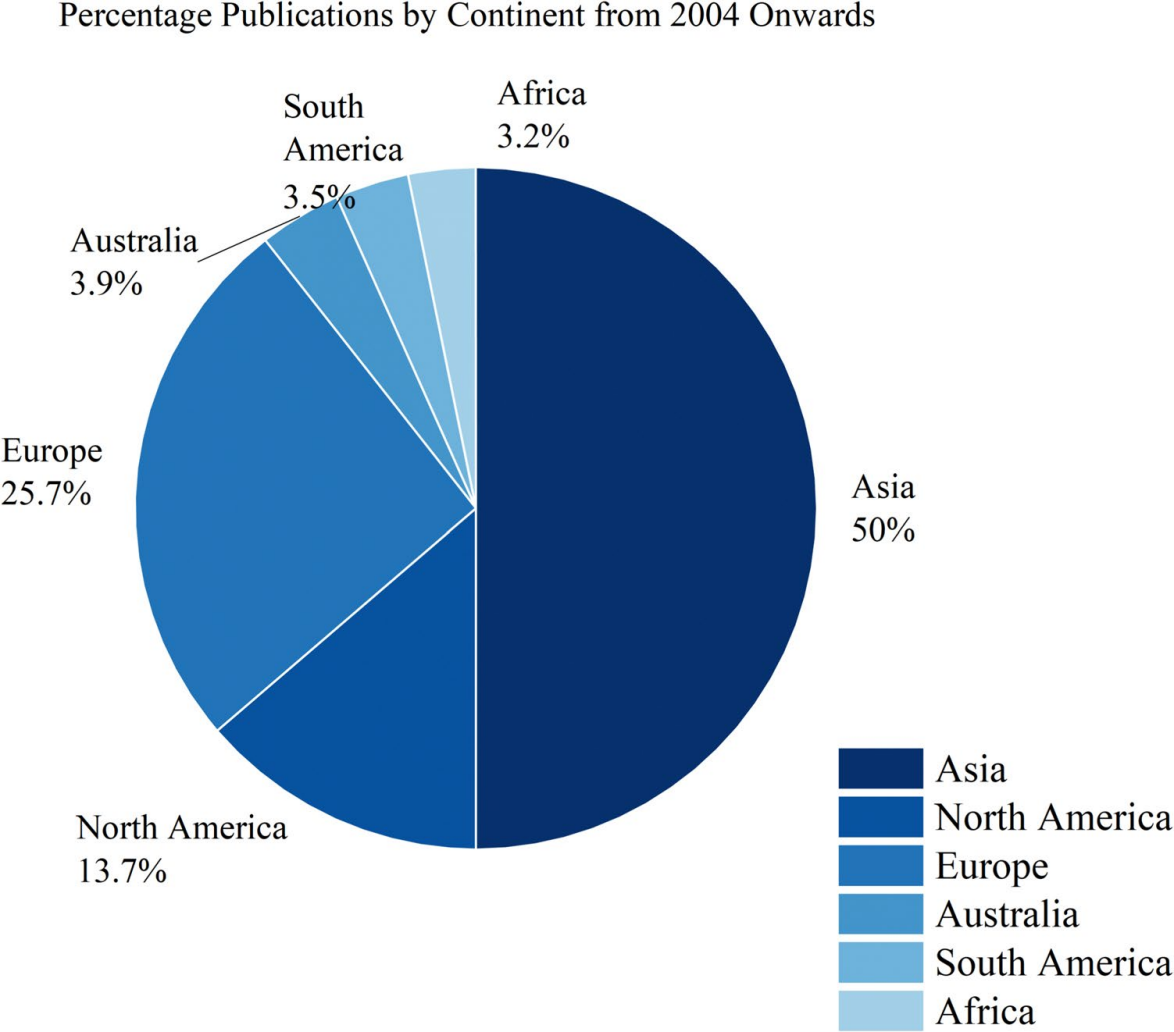
Percentage publications by continent from 2004 onwards

The continent-wise distribution of publications on the treatment of recycled aggregate (2004–2025), as illustrated in Fig. 7, highlights that Asia leads the research output with 20,741 publications (50%), followed by Europe (25.7%) and North America (13.7%). Australia, South America, and Africa contribute smaller shares, ranging between 3.2 and 3.9%. This distribution underscores Asia’s dominant role in advancing sustainable construction research, while Europe and North America also significantly contribute to global efforts.

**Fig. 8:**
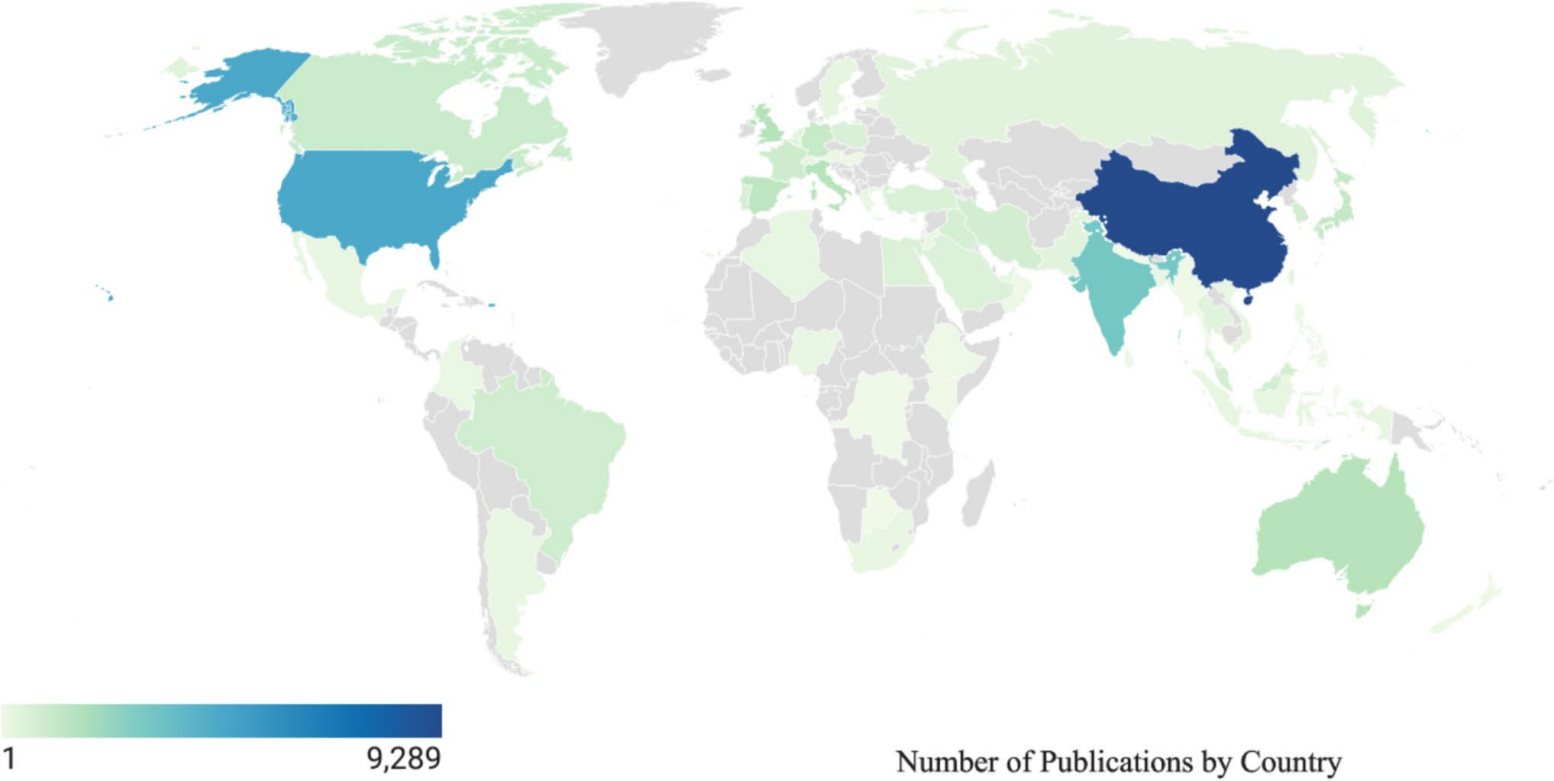
Number of publications by country

Additionally, Fig. 8 presents country-wise publication counts, highlighting leading nations in recycled aggregate treatment research. China is leading, followed by the USA, India, and the UK. Research output is well-distributed across multiple regions, with Asia, Europe, and North America being the dominant contributors. This trend underscores the growing importance of sustainable practices in construction and the increasing global collaboration in this field.

## Significance of review

Despite the considerable number of publications on recycled aggregates and their properties, a critical gap remains in identifying and highlighting the most effective methods for achieving an improvement in the mechanical properties of RC. This paper summarizes studies from individual treatment techniques and provides a detailed discussion on the effects such as aggregate properties (water absorption, density, attached mortar content), mechanical properties (compressive strength, splitting tensile strength, flexural strength, elastic modulus), together with the durability properties (resistance to chloride penetration) and micro-structural observations.

## Methodology

**Fig. 9:**
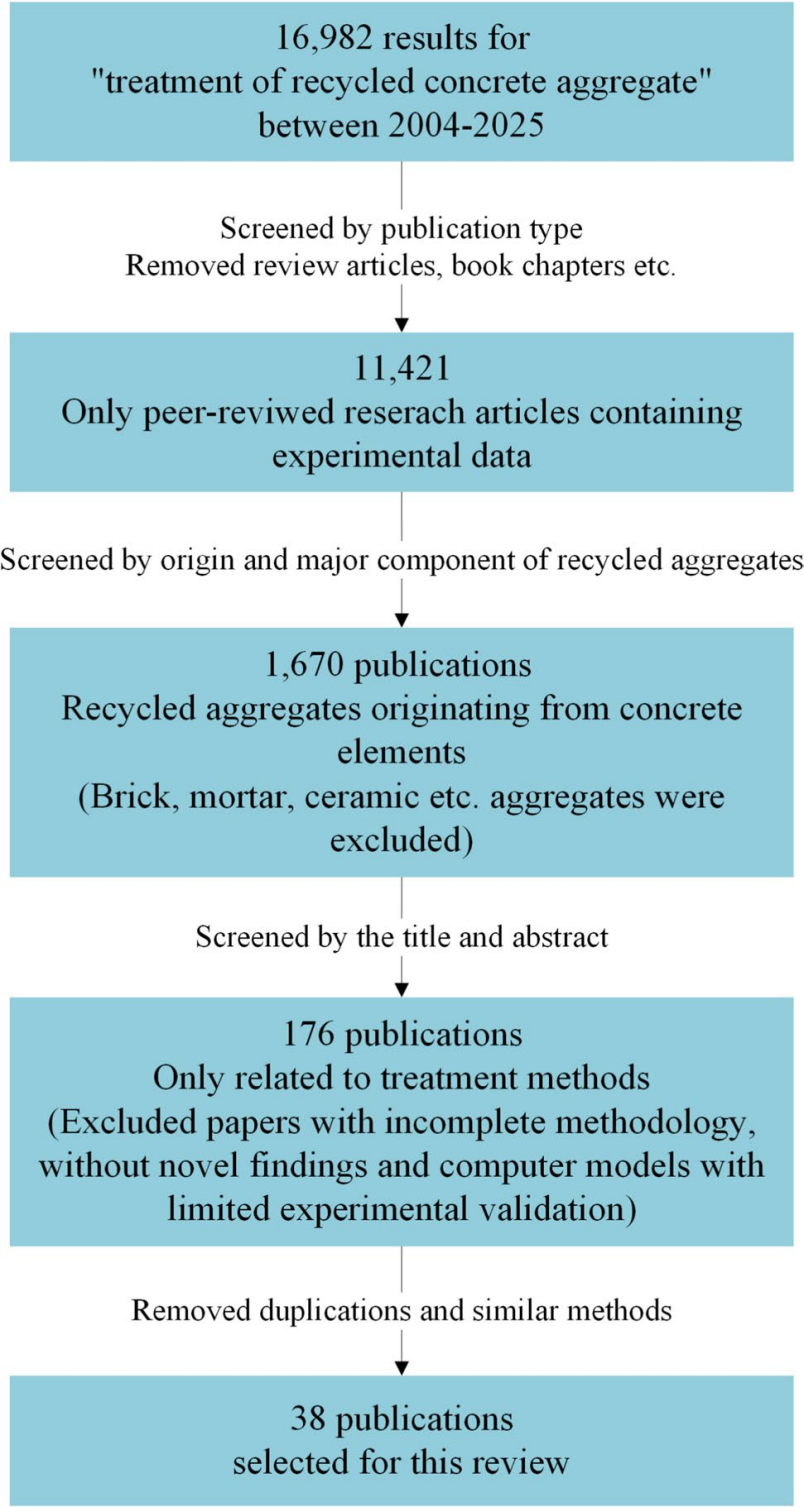
Publication screening process

More than 16,000 publications were initially identified from ScienceDirect, Google Scholar, and Web of Science platforms using search phrases such as “treatment of recycled aggregates,” “treating recycled concrete aggregates,” and “enhancing recycled concrete properties.” To ensure a focused and high-quality review, these studies were filtered based on specific inclusion and exclusion criteria. Figure 9 methodically explains these criteria followed to compile the studies analyzed in this review.

To maintain source diversity, both demolished and laboratory made concrete sources were considered. Among the 38 studies reviewed here, 68% sourced RAs from demolished structures or recycling plants in their local area, and 32% from concrete elements cast in laboratories. To maintain geographic diversity, studies that sourced recycled CDW from across the world, including Japan, India, China, Hong Kong, Singapore, Spain, Türkiye, Cyprus, Saudi Arabia, USA, Canada, Australia, and Brazil are included. To minimize selection bias and ensure a balanced perspective, studies with varying conclusions, including those reporting negative, contradictory and negligible effects of treatment methods are also included. These are highlighted where necessary.

The study reviews the treatment methods, to remove or to strengthen the attached mortar, the effects of the methods on the properties of the RA, a discussion of the reported results, the key conclusions derived from the study, identification of critical gaps in the research and recommendations for future development.

## Treatment methods and their effect on RA

**Fig. 10:**
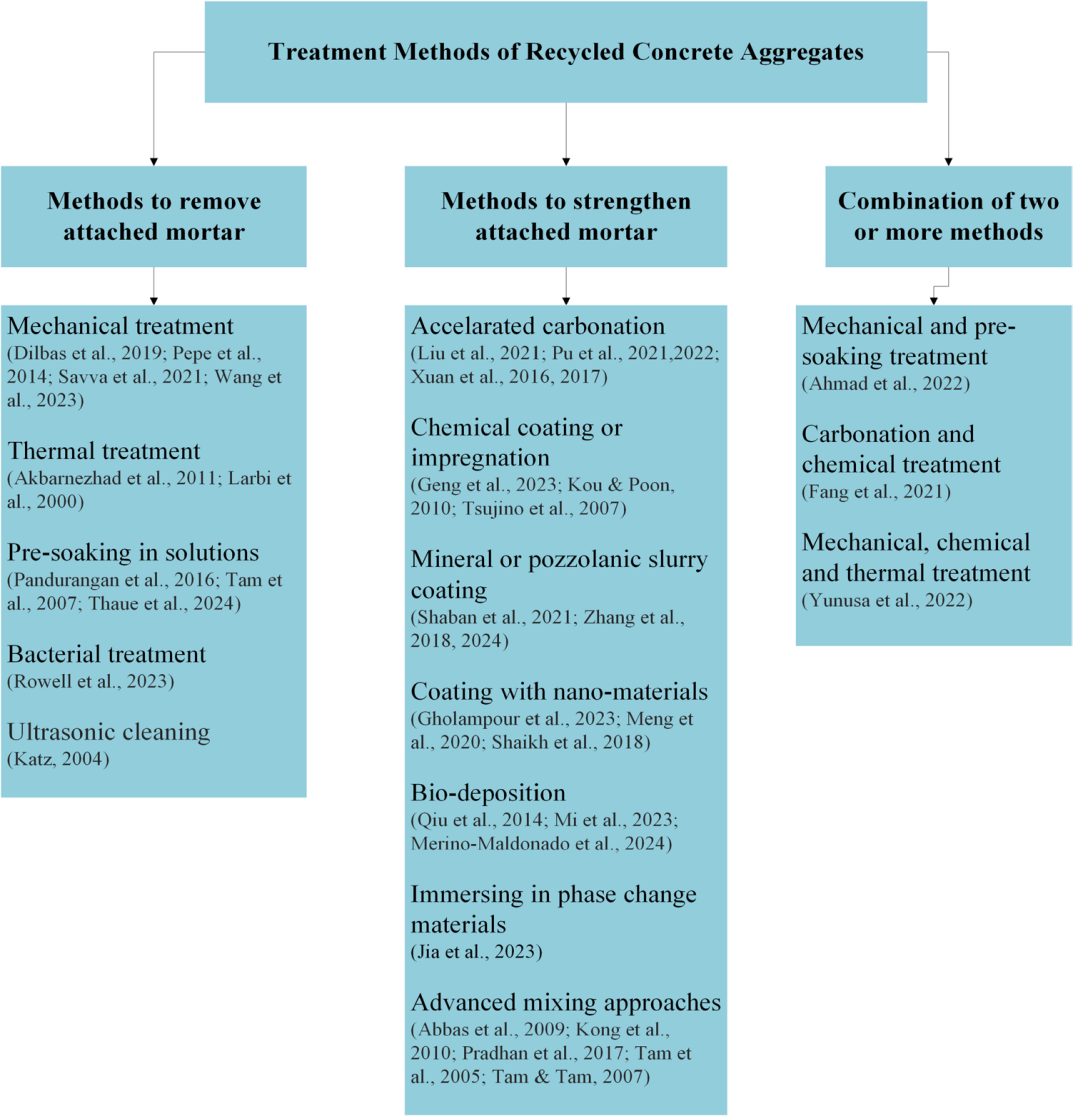
Treatment methods of RA

Primarily, two different types of treatment methods are reported in literature depending on their intended outcome: methods to remove attached mortar and methods to strengthen the attached mortar (Alqarni et al. 2021). The treatment methods are illustrated systematically in Fig. 10. The main techniques to remove attached mortar are mechanical treatment, ultrasonic cleaning, thermal treatment, pre-soaking in solutions, and bacterial treatment. The most common techniques to strengthen the attached mortar are accelerated carbonation, chemical coating or impregnation, mineral or pozzolanic slurry coating, bio-deposition, and coating with nano-materials (Alqarni et al. 2021). A few advanced mixing methodologies namely, two-stage mixing (Tam et al. 2005), triple mixing (Kong et al. 2010), equivalent mortar volume method (Abbas et al. 2009), and particle packing method (Pradhan et al. 2017) have also been explored by researchers to fulfil their objective of enhancing the performance of RC. Literature also shows evidence of efforts to combine two or more treatment methods to obtain RA with lower porosity and RC with elevated structural and durability properties (Ahmad et al. 2022; Fang et al. 2021; Yunusa et al. 2022).

### Methods to remove attached mortar

**Fig. 11:**
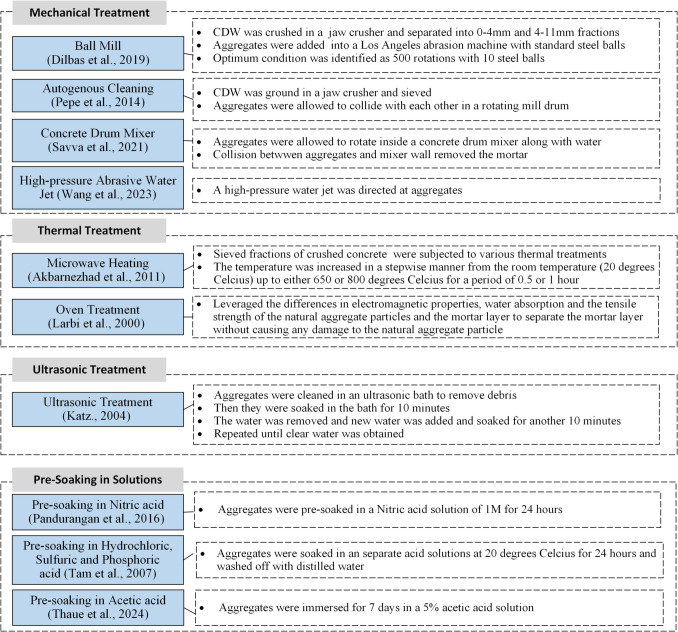
Experimental details of studies incorporating mortar removal methods

Figure 11 provides an overview of the methodologies and the variables investigated in the experimental studies related to mortar removal. Table 2 and Fig. 13 compares the changes in treated recycled concrete aggregates (TRA) properties before and after treatment, while Table 3 summarizes changes in properties of concrete manufactured with TRA when compared with their untreated recycled concrete aggregate (URA) counterparts.

**Table 2:** Percentage changes in aggregate properties due to removal methods

Treatment method	Water absorption (%)			Bulk density (kg/m^3^)			Attached mortar content/mass (%)			Los Angeles coefficient (LAAV) (%)		
	**URA**	**TRA**	**% change**	**URA**	**TRA**	**% change**	**URA**	**TRA**	**% change**	**URA**	**TRA**	**% change**
Ball Mill (Dilbas et al. 2019)	8.95	0.84	** − 90.6%**	1800	2470	**37.2%**	52.50	9.60	** − 81.7%**	55	28	** − 49.1%**
Autogenous Cleaning (Pepe et al. 2014) (19–9.5 mm, 10 min)	4.94	4.01	** − 18.8%**	2634	2358	** − 10.5%**	30.00	15.00	** − 50.0%**	–	–	–
Autogenous Cleaning (Pepe et al. 2014) (19–9.5 mm, 15 min)	4.94	4.09	** − 17.2%**	2634	2328	** − 11.6%**	30.00	15.00	** − 50.0%**	–	–	–
Autogenous Cleaning (Pepe et al. 2014) (9.5–4.75 mm, 10 min)	11.94	6.06	** − 49.2%**	1946	2220	**14.1%**	30.00	15.00	** − 50.0%**	–	–	–
Autogenous Cleaning (Pepe et al. 2014) (9.5–4.75 mm, 15 min)	11.94	5.56	** − 53.4%**	1946	2261	**16.2%**	30.00	15.00	** − 50.0%**	–	–	–
Concrete Drum Mixer (Savva et al. 2021) (10–4 mm)	6.52	4.48	** − 31.3%**	2517	2430	** − 3.5%**	2.95	2.53	** − 14.1%**	32	15	** − 53.1%**
Concrete Drum Mixer (Savva et al. 2021) (20–8 mm)	4.40	4.00	** − 9.1%**	2430	2400	** − 1.2%**	2.98	2.47	** − 17.0%**	32	15	** − 53.1%**
Microwave Heating (Akbarnezhad et al. 2011)	4.20	2.80	** − 33.3%**	2370	2460	**3.8%**	47.00	24.00	** − 48.9%**	–	–	–
Oven Treatment (Larbi et al. 2000)	–	–	–	–	–	–	55.00	5.00	** − 90.9%**	–	–	–
Pre-soaking in Nitric acid (Pandurangan et al. 2016)	4.58	2.90	** − 36.7%**	2450	2590	**5.7%**	29.00	2.00	** − 93.1%**	48.32	–	–
Pre-soaking in Hydrochloric acid (Tam et al. 2007) (20 mm)	1.65	1.45	** − 12.1%**	–	–	–	–	–	–	–	–	–
Pre-soaking in Hydrochloric acid (Tam et al. 2007) (10 mm)	2.63	2.31	** − 12.2%**	–	–	–	–	–	–	–	–	–
Pre-soaking in Sulfuric acid (Tam et al. 2007) (20 mm)	1.65	1.48	** − 10.3%**	–	–	–	–	–	–	–	–	–
Pre-soaking in Sulfuric acid (Tam et al. 2007) (10 mm)	2.63	2.37	** − 9.9%**	–	–	–	–	–	–	–	–	–
Pre-soaking in Phosphoric acid (Tam et al. 2007) (20 mm)	1.65	1.53	** − 7.3%**	–	–	–	–	–	–	–	–	–
Pre-soaking in Phosphoric acid (Tam et al. 2007) (10 mm)	2.63	2.41	** − 8.4%**	–	–	–	–	–	–	–	–	–
Pre-soaking in Acetic acid (Thaue et al. 2024)	6.49	4.54	** − 30.0%**	2260	2330	**3.1%**	–	–	–	–	–	–

**Table 3:** Changes in concrete properties due to removal methods

Property	Treatment method								
	**Mechanical treatment**				**Thermal treatment**		**Pre-soaking in solutions**		
	Ball Mill (Çakır and Dilbas 2021; Dilbas et al. 2019)	Autogenous Cleaning (Pepe et al. 2014)	Concrete Drum Mixer (Savva et al. 2021)	High-pressure Abrasive Water Jet (Wang et al. 2023)	Microwave Heating (Akbarnezhad et al. 2011)	Oven Treatment (Larbi et al. 2000)	Pre-soaking in Nitric acid (Pandurangan et al. 2016)	Pre-soaking in Hydrochloric, Sulfuric and Phosphoric acid (Tam et al. 2007)	Pre-soaking in Acetic acid (Thaue et al. 2024)
**TRA concrete physical and mechanical properties**									
**Water absorption**	24.6–48.8% decrease	N/A	N/A	N/A	N/A	N/A	N/A	N/A	N/A
**Density**	2.6–9.1% increase	N/A	N/A	N/A	N/A	Similar to NA	N/A	N/A	N/A
**28-day compressive strength**	20.1–54% increase	8.8% increase	− 3 and 5.2% increase (for 0.25 and 0.5 w/c) for 25% replacement − 1.9 and 2.1% increase (for 0.25 and 0.5 w/c) for 50% replacement	1.8–26.2% decrease	20% increase for 100% replacement Almost equal to NC for 40% replacement	14% decrease when compared to NC	5.7% increase	Highest increase (14%) is with 25% replacement for H_3_PO_4_	8.7–12.2% increase for 0.4 w/c 11.1–17.7% increase for 0.7 w/c
**28-day splitting tensile strength**	8.7% decrease for 20% replacement 14.5–36.3% increase for 40–60% replacement	10.4% increase	0–2.8% increase for 25% replacement 2.8–20.7% increase for 50% replacement	N/A	N/A	N/A	N/A	N/A	N/A
**28-day flexural strength**	2–6.2% decrease for 20–40% replacement 20.6% increase for 60% replacement	N/A	N/A	N/A	6.4% increase for 100% replacement	N/A	N/A	Highest increase (18.6%) is with 10% replacement for H_3_PO_4_	Approx. 5% decrease for 0.4 w/c Approx. 15% decrease for 0.7 w/c
**28-day elastic modulus**	10.3–38.6% increase	1.85% decrease	N/A	N/A	13.2% increase for 100% replacement	N/A	N/A	Highest increase (20.5%) is with 30% replacement for HCl	N/A
**TRA concrete durability properties**									
**Porosity/Total volume of voids**	N/A	N/A	6.9 and − 0.8% increase (for 0.25 and 0.5 w/c) for 25% replacement 14.4–4.5% decrease for 50% replacement	3.2–8% decrease	N/A	N/A	N/A	N/A	Pore volume and peak diameter decreased. This decrease increased with replacement level
**Resistance to chloride penetration**	50.9–60.5% increase	N/A	32.1 and 18.4% increase (for 0.25 and 0.5 w/c) for 25% replacement 23.7 and 8.9% increase (for 0.25 and 0.5 w/c) for 50% replacement	N/A	N/A	N/A	N/A	N/A	16.1–19.1% increase for 0.4 w/c 4.6–16.7% decrease for 0.7 w/c

#### Mechanical treatment

Various mechanical treatment methods have been applied to remove attached mortar from RA and improve their properties (Fig. 11). The most widely studied techniques are ball milling (Dilbas et al. 2019), autogenous cleaning (Pepe et al. 2014), concrete drum mixing (Savva et al. 2021), and high-pressure abrasive water jets (HAWJ) (Wang et al. 2023).

**Fig. 12:**
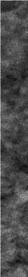
SEM observations of aggregates treated by ball mill (Dilbas et al. 2019)

The ball mill method was conducted using a standard Los Angeles abrasion machine and standard steel balls (Dilbas et al. 2019). To identify the optimum conditions, the number of drum rotations varied from 100 to 500 in increments of 100 and the number of steel balls varied across the range 0, 2, 5, 7, 10, and 12. Increased density and decreased water absorption values were observed with a higher number of steel balls and higher number of rotations. However, SEM observations (Fig. 12) showed that the ball mill induced abrasions, resulting in cracks and discontinuities in the mortar layer. Therefore, the optimum conditions were chosen as 500 rotations and 10 steel balls. This resulted in a noteworthy 81.7% reduction in attached mortar content, and a corresponding 90.6% reduction in water absorption due to the extensive loss of the highly porous mortar layer, together with a 37.2% increase in density. The greatest improvement in the 28-day compressive strength of TRA concrete amongst all removal methods was achieved by ball milling. It was in the range of 20.1–54% for 20–60% replacement in aggregate level. Through visual inspections on concrete specimens, it was noted that that the crack paths traversed through ITZ2 in URA concrete, while for TRA concrete they went through the aggregates themselves. This suggests that the weakest area in URA concrete was the attached mortar layer. The researchers extended the study to investigate concrete durability (Çakır and Dilbas 2021). Here, the concrete mix contained additional silica fume. The rate and depth of water absorption, and chloride ion migration were significantly improved with 10% silica fume.

Mechanical treatment using a drum mixer (Savva et al. 2021) demonstrated the least favorable results, but still produced an improvement in the performance of the treated aggregate. The method resulted in a 19% reduction in attached mortar and a corresponding 9.1–31.3% reduction in water absorption. However, the density reported in this study was the particle density, not the bulk density. Hence, due to the reduction in the voids in the removed mortar, the total volume reduced more than the mass, ultimately increasing the density. The 28-day compressive strength reduced for 0.25 water/cement ratio (w/c) but increased slightly for 0.5 w/c. This phenomenon seems to be an anomaly that does not agree with the general trend of declining compressive strength with increasing w/c. Further investigation is needed to fully understand these anomalies. Even though the mechanical properties of concrete cast with drum mixer TRA were inferior, their durability properties were superior. A 14.4–4.5% decrease in the porosity and a 10.3–23.5% increase in resistivity were obtained with a 50% aggregate replacement level.

A high-pressure abrasive water jet (HAWJ) was used for mortar removal by Wang et al. (2023) due to benefits such as minimal noise, minimal heat generation, and high controllability. However, the intense impact from HAWJ created micro-cracks at the RA-concrete interface which accelerated sulfate attacks and weakened the concrete by 25% in terms of compressive strength. The ultrasonic velocity through concrete decreased by 3.2–8%, which can be directly related to a decrease in porosity.

In a mechanical treatment process known as “autogenous cleaning” (Pepe et al. 2014), RAs were made to collide with each other in a rotating mill drum. This method reduced the mortar content by 50% and in turn decreased the water absorption by 20–50% and increased the density by 3–16%. Mechanical properties such as compressive strength, tensile strength and elastic modulus showed some improvement but not significantly. Durability properties were not explored.

#### Thermal treatment

Thermal treatments, include oven treatment (Larbi et al. 2000) and microwave heating (Akbarnezhad et al. 2011), have been applied as a removal process for the attached mortar layer by inducing the thermal stresses at high temperatures (Fig. 11).

The oven thermal treatment (Larbi et al. 2000) achieved a significant removal of the attached mortar (90%) by heating the RAs to 800 °C for a 2-hour period. The treatment also increased the density to match that of NA. Piezo-response force microscopy (PFM) analysis did not observe any microcracks in aggregates post-treatment, indicating that the treatment had preserved the integrity of aggregates. The investigation did not assess the performance of other crucial TRA properties such as water absorption and density. However, it did report that the compressive strength of concrete declined by 14%. The reason for this loss could be due to the poor bond between aggregates and the cement paste, high water-cement ratio, or poor compaction. microstructural analysis of the ITZs would be necessary to identify the exact cause.

Akbarnezhad et al. (2011) determined the flexural strength and elastic modulus of microwave-TRA to have increased by 6.4% and 13.2% respectively, for 100% replacement. However, the study did not explore any durability properties.

#### Pre-soaking in solutions

**Fig. 13:**
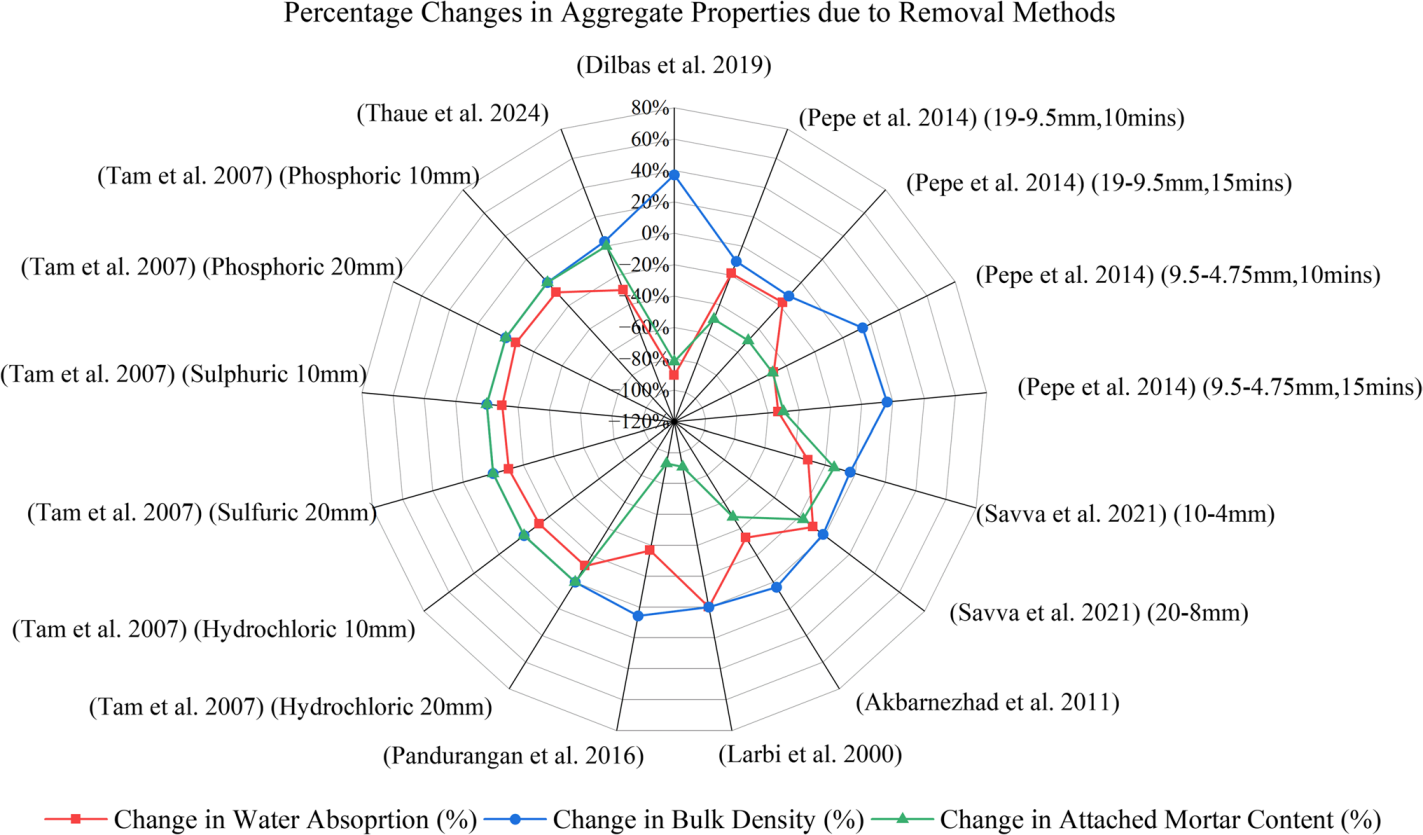
Percentage changes in aggregate properties due to removal methods

Another approach for the removal of the attached mortar from URA is to soak in various solutions (Fig. 11). Pre-soaking RAs in acidic solutions has emerged as an effective method for removing attached mortar. Tam et al. (2007) explored the change in water absorption of RAs when soaked in three different acidic solutions (hydrochloric/HCl, sulfuric/H_2_SO_4_, and phosphoric/H_3_PO_4_). The percentage decreases ranged from 7 to 12%, where HCl and H_3_PO_4_ provided the highest and lowest reduction in water absorption. In other studies with nitric acid (Pandurangan et al. 2016) and acetic acid (Thaue et al. 2024) soaking water absorption of aggregates reduced by 37% and 30% respectively. A 5.4% increase in density was reported with nitric acid-TRAs but was only 1.2–3.1% with acetic acid. The increase in density of nitric acid-TRAs can be attributed to by the 27% decrease in the attached mortar content of URAs (Fig. 13).

Concrete incorporating TRAs treated with solutions showed varying impacts on compressive strength. Tam et al. (2007) reported a 14% increase in 28-day compressive strength when 25% of URAs were replaced with H_3_PO_4_ TRA. Thaue et al. (2024) used different amounts of water (0.4 and 0.7 w/c) in concrete mixes and compared their effect on various concrete properties. An 8.7–12.2% increase (for 0.4 w/c) and a 11.1–17.7% increase (for 0.7 w/c) in compressive strength were observed. However, nitric acid TRA only produced a 5.7% increase. Despite their improvement in compressive strength, the 28-day flexural strength containing acetic-acid treatment aggregates decreased by 5 and 15% for 0.4 and 0.7 w/c, respectively. Moreover, Thaue et al. (2024) reported a significant drop in chloride penetration for both acetic-acid treatment mixes.

#### Bacterial treatment

Bacterial treatment is a novel technique for removing attached mortar from RAs. In this method, silicate-solubilizing bacteria are used to dissolve the attached mortar by releasing acids that break down the cement matrix (Rowell et al. 2023). The results highlighted that these bacteria were able to reduce the weight of the URA by about 10% in 14 days. However, this treatment resulted in an increased rate of concrete degradation due to the release of the acids during the process and the chosen bacterium has links to nosocomial infections, raising concerns about its safety in practical applications.

#### Ultrasonic cleaning

Ultrasonic cleaning involves subjecting RAs to high-frequency sound waves in an water bath to remove the attached mortar (Katz 2004). While this method effectively loosens mortar particles, it caused micro-cracks to form at the aggregate-concrete interface. The 28-day compressive strength of concrete incorporating these TRA improved by 7% in comparison to URA concrete. The presence of microcracks likely prevented greater improvement in strength.

### Methods to strengthen attached mortar

**Fig. 14:**
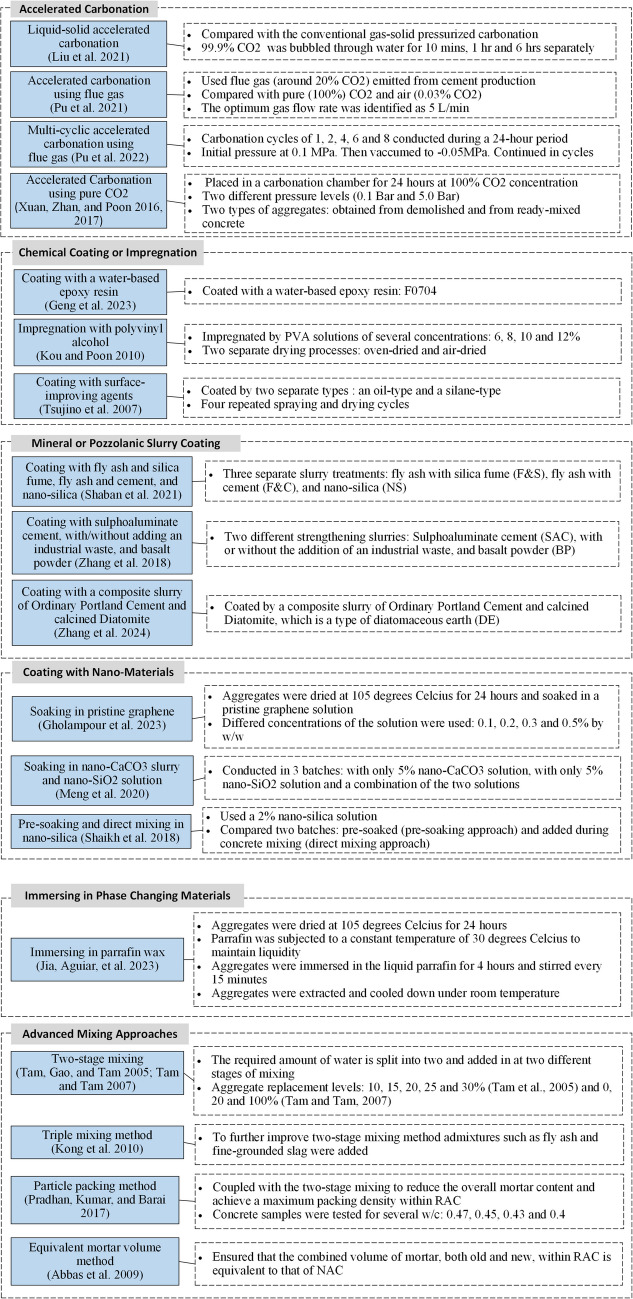
Experimental details of studies incorporating mortar strengthening methods

Experimental details of mortar strengthening methods are composited in Fig. 14. Table 4 and Fig. 19 compare the change in aggregate properties before and after each treatment process. Table 5 and Table 6 present the changes in properties of URA and TRA concrete for strengthening methods.

**Table 4:** Percentage changes in aggregate properties due to strengthening methods

Treatment Method	Water Absorption			Bulk density/Specific gravity			Los Angeles coefficient (LAAV)			Aggregate crushing value (ACV)		
	**URA**	**TRA**	**% change**	**URA**	**TRA**	**% change**	**URA**	**TRA**	**% change**	**URA**	**TRA**	**% change**
Liquid–solid accelerated carbonation (Liu et al. 2021)	9.71	8.55	** − 11.9%**	2080	2140	**2.9%**	–	–	–	–	–	–
Accelerated carbonation using flue gas (Pu et al. 2021)	4.67	4.71	**0.9%**	2653	2649	** − 0.2%**	–	–	–	10.9	11.2	**2.8%**
Accelerated Carbonation using pure CO_2_ (Xuan et al. 2016) (Demolished)	5.50	5.20	** − 5.5%**	2554	2263	** − 11.4%**	–	–	–	26.6	24.5	** − 7.9%**
Accelerated Carbonation using pure CO_2_ (Xuan et al. 2016) (Ready Mix)	6.10	5.00	** − 18.0%**	2249	2301	**2.3%**	–	–	–	27.8	20.6	** − 25.9%**
Coating with a water-based epoxy resin (Geng et al. 2023)	1.70	0.90	** − 47.1%**	–	–	**–**	–	–	–	–	–	–
Impregnation with polyvinyl alcohol (Kou and Poon 2010) (oven-dried)	7.00	3.36	** − 52.0%**	2389.5	2422	**1.4%**	–	–	–	–	–	–
Impregnation with polyvinyl alcohol (Kou and Poon 2010) (air-dried)	7.00	2.00	** − 71.4%**	2389.5	2428.5	**1.6%**	–	–	–	–	–	–
Coating with surface-improving agents (Tsujino et al. 2007) (oil-type)	4.80	3.78	** − 21.3%**	–	–	–	–	–	–	–	–	–
Coating with surface-improving agents (Tsujino et al. 2007) (silane-type)	4.80	1.07	** − 77.7%**	–	–	–	–	–	–	–	–	–
Coating with F&S (Shaban et al. 2021)	7.26	6.09	** − 16.1%**	2.60	2.65	**1.9%**	36.22	25.03	** − 30.9%**	35.81	21.91	** − 38.8%**
Coating with F&C (Shaban et al. 2021)	7.26	3.53	** − 51.4%**	2.60	2.70	**3.8%**	36.22	23.51	** − 35.1%**	35.81	20.41	** − 43.0%**
Coating with NS (Shaban et al. 2021)	7.26	3.21	** − 55.8%**	2.60	2.66	**2.3%**	36.22	24.4	** − 32.6%**	35.81	20.12	** − 43.8%**
Coating with SAC (Zhang et al. 2018)	5.63	5.08	** − 9.8%**	2520	2526	**0.2%**	–	–	**–**	14.56	12.96	** − 11.0%**
Coating with SAC & BP (Zhang et al. 2018)	5.63	4.57	** − 18.8%**	2520	2530	**0.4%**	–	–	–	14.56	11.20	** − 23.1%**
Coating with a composite slurry of OPC and 5% DE (Zhang et al. 2024)	2.32	2.72	**17.2%**	2680	2650	** − 1.1%**	–	–	–	14.6	15.5	**6.2%**
Coating with a composite slurry of OPC and 20% DE (Zhang et al. 2024)	2.32	5.15	**122.0%**	2680	2670	** − 0.4%**	–	–	–	14.6	16.5	**13.0%**
Soaking in nano-CaCO_3_ (Meng et al. 2020)	8.45	8.38	** − 0.8%**	–	–	–	–	–	–	15.579	11.309	** − 27.4%**
Soaking in nano-SiO_2_ (Meng et al. 2020)	8.45	9.68	**14.6%**	–	–	–	–	–	–	15.579	8.962	** − 42.5%**
Soaking in combined nano-CaCO_3_ & nano-SiO_2_ (Meng et al. 2020)	8.45	6.88	** − 18.6%**	–	–	–	–	–	–	15.579	6.915	** − 55.6%**
Immersing in paraffin (Jia et al. 2023b)	7.82	–	–	2134	–	–	–	–	–	–	–	–

**Table 5:** Changes in concrete properties due to strengthening methods (part 1)

Property	Treatment Method						
	**Accelerated carbonation**				**Chemical coating or impregnation**		
	Liquid–solid accelerated carbonation (Liu et al. 2021)	Accelerated carbonation using flue gas (Pu et al. 2021)	Multi-cyclic accelerated carbonation using flue gas (Pu et al. 2022)	Accelerated Carbonation using pure CO_2_ (Xuan et al. 2016, 2017)	Coating with a water-based epoxy resin (Geng et al. 2023)	Impregnation with polyvinyl alcohol (Kou and Poon 2010)	Coating with surface-improving agents (Tsujino et al. 2007)
**TRA concrete physical and mechanical properties**							
**28-day compressive strength**	Approx. 20, 42.2, and 55.6% increase for 10 min, 1 h and 6 h durations	4.5, 7.1, 14.5, 18.6, and 22.3% increase for 20, 40, 60, 80 and 100% replacement	2.3, 10.8, 18.6, 19.5, and 25.2% increase for 20, 40, 60, 80, and 100% replacement	0.6, 10.8, 11, 16.6, and 22.6% increase for 20, 40, 60, 80, and 100% replacement levels (for ready mix RAs) 6.6 and 2.3% increase when pressure was increased from 0.1 to 5 Bar for demolished and ready-mix RAs	4% increase	1.1 and 5.6% increase for oven and air-dried RAs	Approx. 6% increase and 40% decrease for oil and silane-type (for 60% w/c) Approx. 12% and 41% decrease for oil and silane-type (for 40% w/c)
**28-day splitting tensile strength**	N/A	N/A	N/A	N/A	2.5% increase	(− 6.5) and 1.1% increase for oven and air-dried	N/A
**28-day flexural strength**	N/A	2, 9.1, 15.4, 19.4, and 23.5% increase for 20, 40, 60, 80, and 100% replacement	4.1, 11.6, 17.9, 22.2, and 26.5% increase for 20, 40, 60, 80, and 100% replacement	4.9, 7.4, 12.4, 17.2, and 28.7% increase for 20, 40, 60, 80, and 100% replacement levels (for ready mix RAs) 0.9 and 2.4% increase when pressure was increased from 0.1 to 5 Bar for demolished and ready mix RAs	N/A	N/A	N/A
**TRA concrete durability properties**							
**Drying Shrinkage**	N/A	3.2–20.4% decrease	N/A	23–25.2% decrease for ready mix RAs No decrease for demolished RAs	N/A	Approx. 13 and 10% decrease for oven and air-dried	Least shrinkage exhibited by oil-type
**Resistance to chloride penetration**	N/A	N/A	N/A	8.8, 4.4, 13.6, 24.5, and 36.4% increase for 20, 40, 60, 80, and 100% replacement levels (for ready mix RAs) 15.4 and 11.5% increase when pressure was increased from 0.1 to 5 Bar for demolished and ready-mix RAs	N/A	Approx. 35 and 33% increase for oven and air-dried	N/A
**Creep strain**	N/A	N/A	N/A	N/A	N/A	N/A	No effect on creep strain for oil-type Substantially high creep strain for silane-type

**Table 6:** Changes in concrete properties due to strengthening methods (part 2)

Property	Treatment Method										
	**Mineral or Pozzolanic Slurry Coating**			**Coating with Nano-Materials**			**Immersing in phase changing materials**	**Advanced Mixing Approaches**			
	Coating with F&S, F&C, and NS (Shaban et al. 2021)	Coating with SAC & BP (Zhang et al. 2018)	Coating with a composite slurry of OPC and DE (Zhang et al. 2024)	Soaking in pristine graphene (Gholampour et al. 2023)	Soaking in nano-CaCO_3_ slurry and nano-SiO_2_ solution (Meng et al. 2020)	Pre-soaking and direct mixing in nano-silica (Shaikh et al. 2018)	Immersing in paraffin (Jia et al. 2023b)	Equivalent mortar volume method (Abbas et al. 2009)	Triple mixing method (Kong et al. 2010)	Particle packing method (Pradhan et al. 2017)	Two-stage mixing (Tam et al. 2005; Tam and Tam 2007)
** TRA concrete physical and mechanical properties**											
**28-day compressive strength**	Approx. 75, 60 and 63% increase for F&S, F&C and NS	19 and 32.5% increase for SAC an BP	Increased but the percentage increase decreased with the DE Dosage Maximum and minimum increases were 20 and 8% for 5 and 20% doses	9, 21, 6 and 2% increase for 0.1, 0.2, 0.3 and 0.5% w/w 0.2% w/w concrete strength is almost equal to NA	33–40% increase for combined treatment 71% increase for treatment with dispersant	20 and 25% increase for pre-soaking and direct mixing	16% decrease	6% decrease for RAs sourced from Montreal 2% increase for RAs sourced from Vancouver	Approx. 30% increase compared to normal mixing	Decreased compared to conventionally graded RA	9.4, 8.9, 21.2, 20.6 and 13.9% increase for 10, 15, 20, 25 and 30% replacement
**28-day splitting tensile strength**	N/A	N/A	N/A	N/A	N/A	N/A	N/A	N/A	N/A	6.3% increase compared to conventionally graded RA	N/A
**28-day flexural strength**	N/A	N/A	N/A	N/A	N/A	N/A	23% decrease	N/A	N/A	24.4% increase compared to conventionally graded RA	N/A
**TRA concrete durability properties**											
**Drying Shrinkage**	N/A	N/A	N/A	N/A	N/A	N/A	N/A	N/A	N/A	N/A	Increased with time but decreased compared to normally mixed concrete
**Resistance to chloride penetration**	Approx. 48, 22 and 24% increase for F&S, F&C and NS	Increased with treatment and time Higher increase is with BP	Increased but the percentage increase decreased with the DE dosage	N/A	N/A	Improved more with pre-soaking than direct mixing by 8%	N/A	Approx. 10% increase	Approx. 40% increase compared to normal mixing	N/A	Inconsistent change with time and when compared to normally mixed concrete
**Creep strain**	N/A	N/A	N/A	N/A	N/A	N/A	N/A	N/A	N/A	N/A	Increased with time but decreased compared to normally mixed concrete

#### Accelerated carbonation

Accelerated carbonation has been used by many experimental studies to introduce CaCO_3_ into the attached mortar layer of URAs, effectively filling the voids and strengthening the aggregate (Figure 14). Xuan et al. (2016) observed a steady increase in concrete density when replacing URAs with TRAs, with a maximum 1.5% at 100% replacement. However, increasing the carbonation pressure from 0.1 Bar to 5.0 Bar only yielded a minor density increase of 0.5%. The 28-day compressive strength of concrete with TRAs was enhanced by 22.6% at 5.0 Bar. A similar pattern of results was observed with both static modulus of elasticity and flexural strength of concrete. An extension of the experiment to the durability properties of concrete were conducted by Xuan et al. (2017). The results highlighted that the treatment process decreased the water absorption of TRA concrete. It is noticeable that the bulk electrical conductivity, chloride ion permeability and gas permeability were also decreased by 15.1%, 36.4%, and 42.4% respectively.

Pu et al. (2021) identified flue gas containing 20% CO_2_ as the most suitable agent for efficient carbonation, when also considering environmental impact and economic viability. A further study (Pu et al. 2022) indicated that six carbonation cycles were the optimal number for enhancing performance, reducing TRA porosity by 23.33%, increasing compressive strength and flexural strength by 25.2% and 26.5% compared to URA concrete.

In a novel liquid–solid carbonation process, Liu et al. (2021) demonstrated that a 10-min treatment was more effective in enhancing the properties of RA, such as density and water absorption, than longer treatments.

#### Chemical coating or impregnation

Chemical coating and impregnation have been explored as methods to enhance the properties of RAs (Fig. 14). Two surface-acting agents, silane-based (containing 68–72% water) and oil-based (containing 85–95% paraffin) were explored by Tsujino et al. (2007). Aggregates treated with the silane-type agent had a significant reduction in water absorption compared to the oil-type RA. Thus, it is deduced that the silane-type agent had penetrated further into the mortar pores and strengthened the aggregates to a greater degree. The compressive strength declined for both oil and silane aggregates with at 0.4 w/c (by around 40%) and gave a similar decrease for silane with 0.6 w/c, indicating that w/c had little effect on the utilization silane treated aggregates. Nonetheless, the strength did improve for mixes including oil-type TRAs at 0.6 w/c and has almost matched that of NA. The strength reduction with 0.4 w/c could be due to the formation of a film around aggregates, causing a reduction in bond strength between the aggregate and cement matrix. Enhancement in creep strain and drying shrinkage was also superior with the oil-type agent due to stronger bond properties and a lower peeling effect than silane-type agent.

The use of polyvinyl alcohol (PVA) achieved a considerable improvement with regards to water absorption (Kou and Poon 2010). This effect was observed to be more prominent with air-dried aggregates than those oven-dried. The particle density, however, only increased by around 2%. This could be because PVA only covered the outer surface of mortar and had not penetrated to the inside. Hence, the PVA restricted the ingress of water successfully but had not fully coated the pores and thus did not increase the density significantly. The compressive strength only improved by 1–5%. This further reinforces the hypothesis that PVA has only formed a cover around the mortar surface but has not penetrated inside. The resistance to chloride penetration and drying shrinkage were also enhanced, which would further support the premise that the PVA only coats the mortar surface.

Epoxy-resin treated aggregates exhibited a slightly lower water absorption and higher compressive and splitting tensile strength values than their untreated counterparts (Geng et al. 2023). The study did not assess the durability properties of the treated aggregate concrete.

#### Mineral or pozzolanic slurry coating

Many mineral and pozzolanic slurry coatings have been explored for their potential to enhance the properties of RAs (Fig. 14). Zhang et al. (2024) incorporated a mix of OPC (Ordinary Portland Cement) and DE (diatomaceous earth) as the coating and observed an exceptional increase in water absorption of 122%, with a dose of 20% DE. However, even with a minimal dose of 5% DE, the water absorption was elevated by 17%, which is greater than all other methods in this review. DE is known to possess a highly porous structure with a high water demand itself (Ergun 2011), therefore it can be deduced that water absorption will be escalated when coating aggregates. The crushing value also rose with the DE dosage due to the ease of peeling of the thicker DE coating from the RA particles under compression. The compressive and splitting tensile strength improved and exceeded those of untreated RC. This is due to the silica present in DE reacting with Ca (OH)_2_ to form additional C-S–H. With the increase in DE dose, compressive and splitting tensile strengths weakened. Many DE particles remain unreacted with a high proportion of DE. There is also an increase in voids due to evaporation of water. This also accounts for a drop in the resistance to chloride diffusion with increased DE dosage. Overall, the research concluded that a low dosage of DE mixed with OPC can effectively work as an efficient surface treating agent to enhance the performance of the RAs.

In the experiment conducted by Shaban et al. (2021) assessing a range of additives the water absorption of aggregates was reduced most by nano-silica (NS), fly ash and cement (F&C), and fly ash and silica fume (F&S), in that order. In addition to filling the voids by the pozzolanic slurries, they also formed a thin layer of pozzolan around the RAs, which acted as a shield to prevent water ingress. This layer was more prominent with NS than with F&S. Coating with F&S, F&C, and NS also performed well in terms of improving the compressive strength of concrete at 28 days. F&S slurry exhibited superior strength enhancement. Chloride penetration was also hindered by the three slurries. Therefore, it was concluded that all three slurries improved the performance of URAs. However, with regards to cost effectiveness and CO_2_ emissions, the most feasible slurry is F&S (Shaban et al. 2021).

Zhang et al. (2018) studied the effect of coating with sulphoaluminate cement (SAC), with/without an industrial waste, and basalt powder (BP). A significant reduction in water absorption and ACV were reported. The reduction was more effective with the addition of BP. A similar trend was observed with an enhancement of 28-day compressive strength and chloride resistance.

#### Bio-deposition

**Fig. 15:**
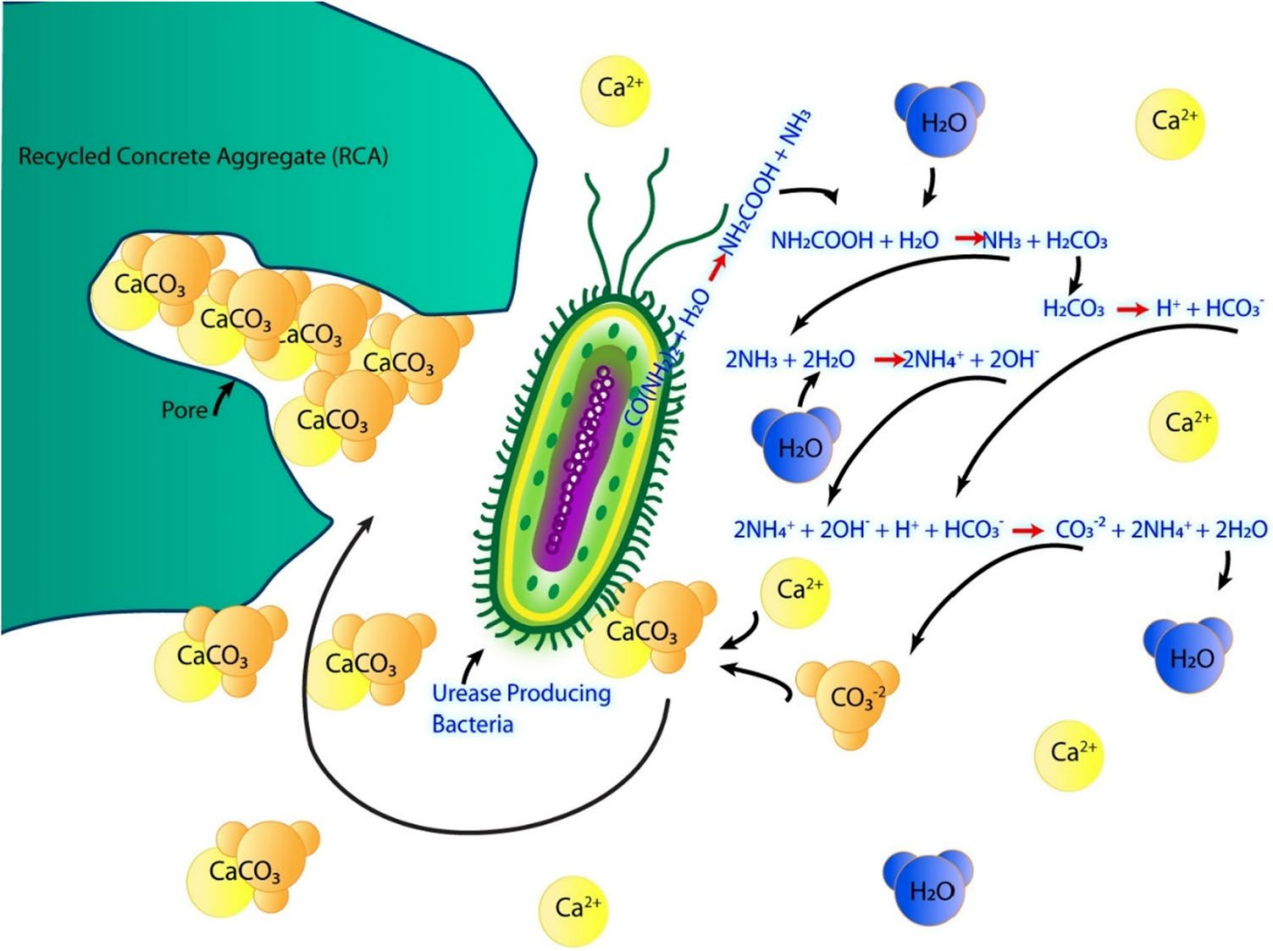
Bio-deposition of CaCO_3_ in RA (Tam et al. 2021)

Bio-deposition, a novel treatment method introduced by Qiu et al. (2014), uses microbial carbonate precipitation to enhance the properties of URA. It employs miro-organisms, in this case *Sporosarcina pasteurii* DSM 33 bacteria, to precipitate CaCO_3_ crystals among URA pores. The mechanism is elaborated in Fig. 15. Under optimum conditions, RA weight increased by 1.03% and reduced the water absorption by 15%. Energy-dispersive X-ray spectroscopy (EDX) analysis carried out on TRA surfaces showed that the type of mineral precipitates is largely determined by the rate of precipitation.

In a comparative study, Mi et al. (2023) investigated both microbial-induced and enzyme-induced carbonate precipitation treatments. Using *Sporosarcina pasteurii* DSM 33 bacteria and urease enzyme, the 7-day compressive strength of TRA concrete surpassed the 28-day compressive strength of URA concrete. The average increases of compressive strength for bacteria and enzyme TRA concrete were 5.8% and 7.8%, respectively. The splitting tensile strength also improved with the treatments. Durability tests showed TRA concrete samples had a higher chloride resistance than URA, with the enzyme-TRA performance superior to that of bacteria-treated.

Another bio-deposition method investigated by Merino-Maldonado et al. (2024) involved the controlled deposition of biogenic silica, using single-celled microalgae named diatoms to produce biogenic silica. They behave as surface modification agents by attaching to surfaces by secreting extracellular polymeric substances which in turn form a layer of silica around that surface. Experimental results indicated that biogenic silica treatment enhanced the 360-day compressive strength by 8% for indoors and 4% and for outdoor TRA, compared to URA. This observation was justified through the Ultrasonic Pulse Velocity technique. Indoor TRA concrete also demonstrated a superior resistance to chloride ion diffusion than its outdoor counterpart. However, both samples had a higher chloride resistance, freeze–thaw resistance, and electrical resistivity than concrete with URAs.

#### Coating with nano-materials

**Fig. 16:**
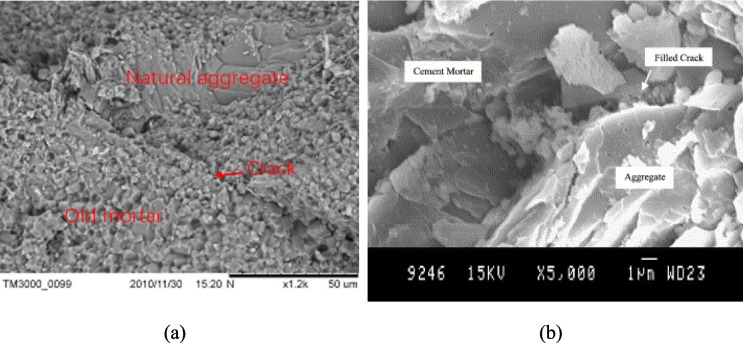
(**a**) SEM image of aggregates treated by combined treatment (Meng et al. 2020). (**b**) SEM of concrete made using two-stage mixing (Tam et al. 2005)

**Fig. 17:**
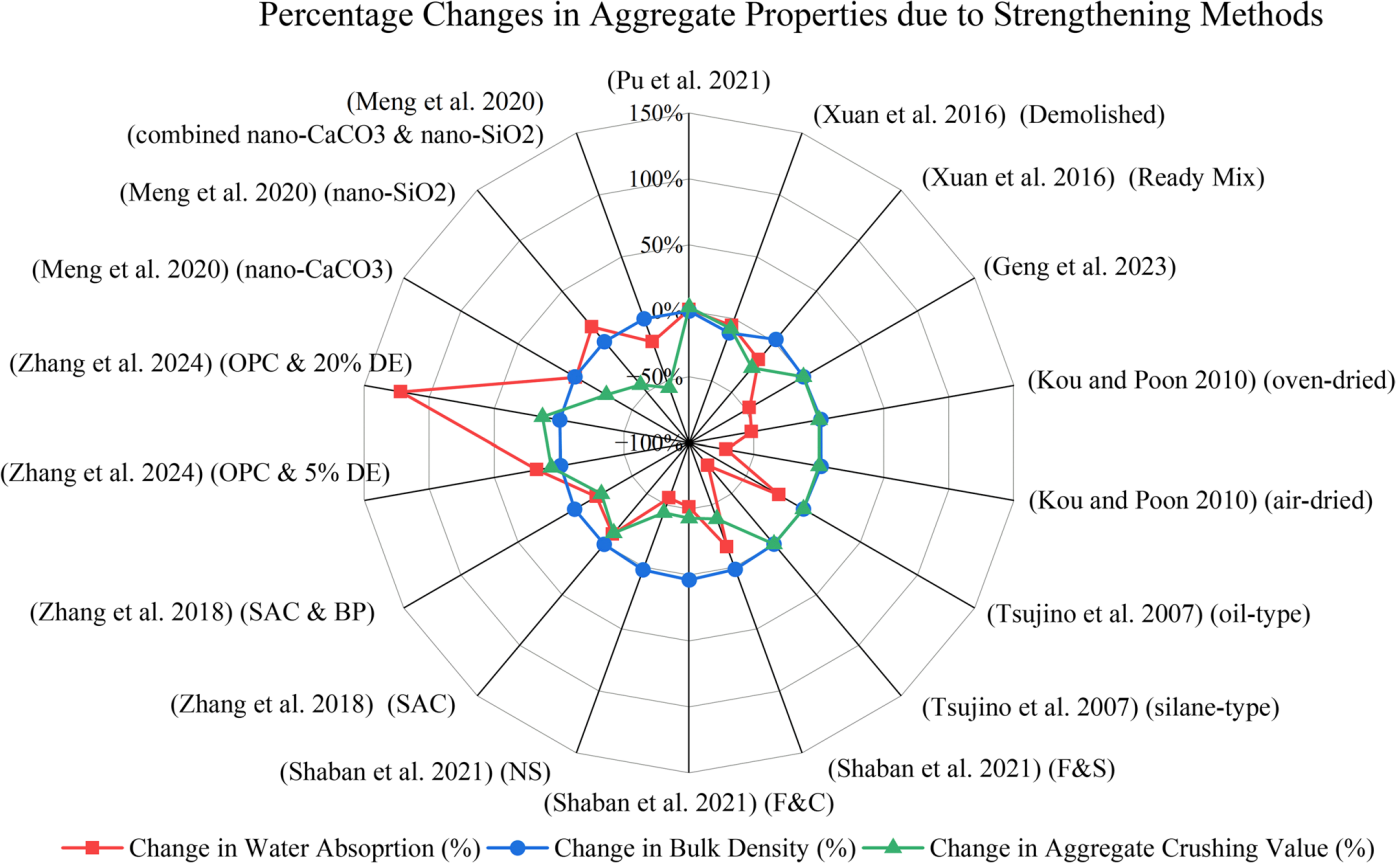
Percentage changes in aggregate properties due to strengthening methods

Nano-materials, such as graphene, nano-CaCO_3_, and nano-SiO_2_, have also been used to improve the properties of RAs (Meng et al. 2020). Among the methodologies used, pre-soaking in a combined treatment of nano-CaCO_3_ and nano-SiO_2_ has proven to be most effective in terms of reducing water absorption of URAs. Using nano-SiO_2_ in solitude worsened the water absorption due to the formation of a thin film on the aggregates. The combined treatment halved the total pore area of the specimens, rendering it the most effective treatment in the study. SEM analysis indicated the settlement of tiny crystals on the surface of RAs filling cracks and discontinuities (Fig. 16(a)). Due to the enhanced dispersibility of the nano-materials, the slurry containing nano-CaCO_3_ with a dispersant, exhibited a significant improvement in compressive strength. Hence, it is deemed the most suitable of the nano-slurries (Fig. 17).

In the study by Gholampour et al. (2023), graphene-TRAs showed a peak reduction in water absorption at a 0.3% w/w dosage. This was attributed to the non-uniformly distributed agglomeration of nano-particles within the pores. Regarding the concrete mechanical properties, 0.2% w/w dosed graphene-TRA, almost equaling that of NA at 28-day compressive strength. However, at elevated dosages, pristine graphene layers cluster together on the surface of RAs, obstructing their dispersion and effective distribution within the matrix.

Shaikh et al. (2018) reported compressive strength improvements of 20 and 25% of pre-soaked and directly mixed nano-silica TRA concrete. The durability tests noted that the volume of permeable voids reduced similarly with pre-soaked and directly mixed aggregate concrete, but water absorption was lower for the pre-soaked aggregate concrete (Shaikh et al. 2018).

#### Immersion in phase changing materials

Immersing recycled aggregates in phase-changing materials (PCM) is a relatively new approach with potential benefits for thermal regulation in concrete (Jia et al. 2023a). PCMs are substances that absorb and release large amounts of latent heat during phase transitions, typically from solid to liquid and vice versa, without a significant change in temperature. This property makes PCMs highly effective for thermal energy storage and temperature regulation in buildings and infrastructure. Research has utilized various PCMs such as polyethylene glycol, diatomite, ceramiste, and paraffin among many others (Jia et al. 2024). A study by Jia et al. (2023b) immersed RAs in liquid paraffin as the treatment method. The PCM filled the pores of the RA, lowering the overall water absorption during mixing. As a result, this adjustment led to a more controlled and reduced water demand, improving the workability. A reduction in the dry density of RC is also recorded. However, it was observed to cause a 16% reduction in 28-day compressive strength and a 23% decrease in 28-day flexural strength when compared to an untreated counterpart. This is likely due to weakened interfacial bonding and changes in the aggregate microstructure. Research on aggregate property modifications and long-term concrete durability is limited, and this treatment method is still in its early stages, requiring extensive exploration before it can be considered for practical applications in construction.

#### Advanced mixing

One of the most notable advanced mixing approaches developed is the “two-stage mixing” method introduced by Tam et al. (2005) (Fig. 16). This novel approach has provided a considerable improvement in compressive strength and durability properties (drying shrinkage, creep strain) (Tam et al. 2005; Tam and Tam 2007). It is attributed to the initial addition of water, which forms a thin layer of cement slurry that fills the voids in the attached mortar of RAs, while the later addition of water facilitates the formation of concrete paste. This process was verified through SEM imagery (Fig. 16(b))*.*

**Fig. 18:**
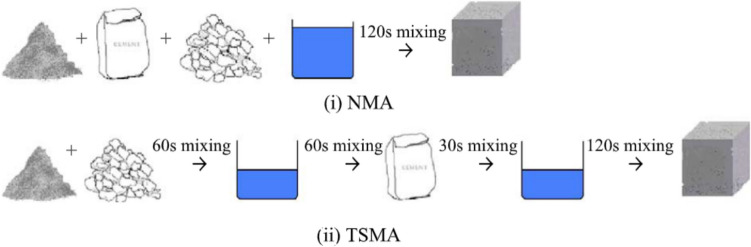
Process of normal mixing approach and two-stage mixing approach (Tam et al. 2005)

**Fig. 19:**
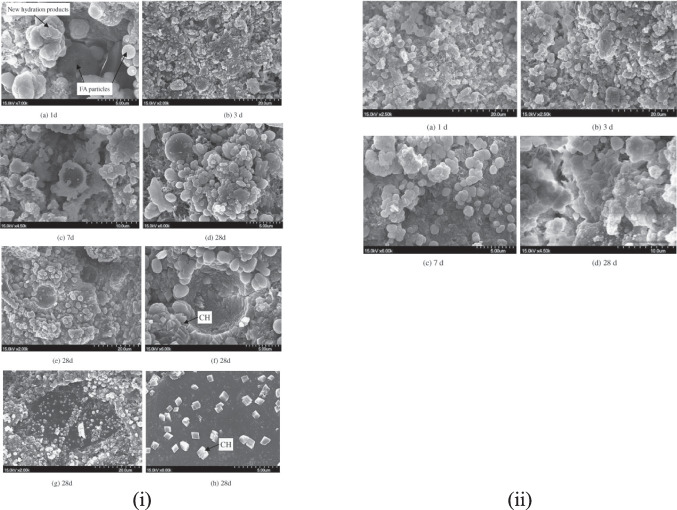
SEM imagery of process of triple mixing with (i) fly ash and (ii) slag (Kong et al. 2010)

The triple mixing method (Kong et al. 2010) improved results from two-stage mixing even further (Fig. 18). The addition of highly reactive fine particles of fly ash filled the RA pores and reacted with unconsumed Ca(OH)_2_ to for new hydration products (Fig. 19(i)(a)–(c)). However, some of the unreacted fly ash particles and Ca(OH)_2_ had accumulated around the ITZ at 28 days, weakening the ITZ, (Fig. 19(i)(d), (h)). This did not, however, occur with slag particles (Fig. 19(ii)(d)). This gave rise to better performance with slag incorporated concrete than fly ash.

The “particle packing method” combined with the two-stage mixing was investigated by Pradhan et al. (2017). For w/c 0.45, the compressive strengths at 7, 28, and 90 days were inferior to conventionally graded RA. Despite this, splitting and flexural tensile strengths did show improvement, while no significant difference was observed in the modulus of elasticity.

Out of advanced mixing methodologies, the equivalent mortar volume (Abbas et al. 2009) method had the least impact, with minor improvements in compressive strength observed. The study lacked microstructural data to interpret mechanisms underpinning this minor change.

### Combination methods

Based on the results from individual RA treatment methods discussed previously, research has expanded to explore techniques involving combinations of two or more such techniques to further enhance RA performance.

Ahmad et al. (2022) compared mechanical abrasion using a Los Angeles test machine with a combination of mechanical abrasion followed by pre-soaking in a 10% sodium silicate solution. The combination method resulted in a notable improvement in compressive strength during the entire test period (7 to 90 days), with further enhancements observed in splitting tensile strength. The sodium silicate treatment also reduced water absorption by 4% and chloride penetration by 24%. The corrosion potential exceeded the threshold values for corrosion initiation for untreated and abrasion only treated RA concrete at 90 days after exposure to a chloride solution. However, even after 140 days, the samples that underwent the combined treatment remained passive state. This effect was attributed to the densification of the concrete matrix. The formation of additional C-S–H is produced by the reaction between sodium silicate and Ca(OH)_2_. 

Fang et al. (2021) explored the effectiveness of spraying a Ca^2+^-rich solution on URAs followed by a flow-through carbonation process. The samples were separated into two batches. One batch was sprayed with wastewater generated from a local concrete batching plant enriched with Ca^2+^ and carbonated (combined treatment), and the other was sprayed with regular tap water and carbonated (carbonation treatment). In comparison with URAs, carbonation-TRAs demonstrated a higher density and lower water absorption. However, combined-TRAs further enhanced both properties. This was due to the introduction of foreign Ca^2+^ ions, which led to the production of more carbonation products, further reducing porosity. Compressive strength tests showed combined-TRAs contributed to a slight increase in comparison to concrete with carbonation-TRAs at both 7 days and 28 days. The compressive strength of combined-TRA concrete was similar to that of NA at 7 day and even surpassed it at 28 days. The depth of chloride penetration, density, and water absorption in concrete with the combined treatment was less than carbonation treatment.

Another combined method which involved mechanical, chemical and thermal treatment was conducted by Yunusa et al. (2022). This multi-step process involved mechanical crushing by using a Los Angeles test machine, immersion in HCl and H_2_SO_4_ solutions at various concentrations (1 M, 2 M, 4.5 M, and 5 M, where M indicates molarity) for 48 hours. The samples were then heated at 400 °C for 2 hours and allowed to cool. The study found that the lowest water absorption was obtained with 2.5 M H_2_SO_4_ acid treatment. The highest mass loss in the TRA samples after freezing and thawing was for 5 M HCl TRA, followed by 4.5 M HCl wherein the most stable was obtained with 2.5 M HCL. With H_2_SO_4_ TRA, the most stable was found in 4.5 M followed by 2.5 M, 1 M then 5 M, which shows no direct relationship with molarity. The aggregate crushing value of TRAs increased with higher concentration of acidic solutions, with H_2_SO_4_TRA showing lower values than those treated with HCl, possibly due to gypsum formation in the voids.

Wenzel et al. (2024) utilized a combined thermal/mechanical and accelerated carbonation treatment. The initial thermal/mechanical process reduces porosity by removing loose mortar and weaker sections of the aggregate. This is then followed by accelerated carbonation, which fills the residual pores and strengthens the ITZ. This combined approach enhances mechanical strength and density while increasing durability in harsh environmental conditions due to reduced water absorption.

### Effectiveness of treatment methods

Removing the attached mortar focuses on addressing the high porosity and water absorption, which often lead to lower density and weaker mechanical properties compared to natural aggregates. Techniques like ball mill crushing and autogenous cleaning are commonly used to reduce the porous mortar layer. This approach helps improve density and compressive strength, making RC closer in performance to NC. As a result, it enhances the aggregates’ suitability for structural applications, especially where high strength and low porosity are critical. However, mechanical removal methods can create micro-cracks in the aggregate, as reported by Dilbas et al. (2019) in ball milling and by Katz (2004) in ultrasonic cleaning. The presence of micro-cracks may reduce long-term durability, which is particularly problematic for applications subjected to cyclic loading or harsh weather conditions, as these micro-cracks can serve as initiation points for freeze–thaw damage and sulphate attack. These methods are also energy-intensive, raising the environmental and economic costs. Consequently, removal treatments may be most suitable for projects prioritizing high mechanical strength without a strong emphasis on durability under environmental stresses.

Strengthening attached mortar treatments work by reinforcing the existing mortar layer, which helps improve long-term durability. Methods such as accelerated carbonation (Liu et al. 2021; Pu et al. 2022, 2021; Xuan et al. 2016, 2017), nano-silica coatings (Meng et al. 2020), and polyvinyl alcohol coatings (Kou and Poon 2010) fill voids and strengthen the ITZs. These treatments enhance the aggregate resistance to environmental degradation, such as chloride penetration and freeze–thaw cycles, making RAs more durable for use in aggressive environments, including marine structures or infrastructure exposed to de-icing salts. However, strengthening treatments may not significantly reduce water absorption or overall porosity, as they primarily improve surface durability rather than internal structure. Some treatments, particularly chemical coatings, focus on covering surface pores, leaving deeper voids unaffected. Consequently, they do not penetrate deeply enough to fill internal voids or effectively reduce overall porosity, which limits their impact on bulk water absorption. While these coatings enhance surface durability and resilience to environmental degradation, they may not reduce internal porosity as effectively as treatments such as accelerated carbonation. Carbonation is particularly effective as it penetrates deep into the aggregate, filling internal voids and enhancing bulk density and compressive strength. Accelerated carbonation promotes the formation of CaCO₃ and decalcified C-S–H, where the decalcified C-S–H in RA reacts with Ca(OH)₂, thereby increasing concrete strength (Leemann et al. 2023). This internal strengthening results in a denser, more durable RA, boosting load-bearing capacity and making carbonation highly advantageous for structural applications. By reducing both surface and internal porosity, carbonation lowers water absorption throughout the aggregate, extending beyond just the surface.

Combined treatments leverage both mortar removal and strengthening processes to achieve enhanced mechanical properties and durability. The multi-step process of mechanical, chemical, and thermal treatments introduced by Yunusa et al. (2022) enhanced RA stability, particularly in freeze–thaw conditions. It shows strong potential for applications in severe environmental conditions, such as sulphate-rich soils or fluctuating temperature environments.

## Practical applications of treatment methods

### Structural applications in concrete

Coarse aggregates play a crucial role in structural concrete, and RAs have been successfully integrated into infrastructure worldwide, with notable examples including Germany’s Vilbeler Weg and Waldspirale, Hong Kong’s Wetland Park, Shanghai’s commercial building, research laboratory building of Kajima Technical Research Institute (Xiao et al. 2022). These cases highlight the potential for RAs in both structural and non-structural applications, provided they meet certain performance criteria. The classification of RAs based on Australian standards into heavyweight (particle density exceeding 3200 kg/m^3^), normal weight (particle density between 2100 kg/m^3^ and 3200 kg/m^3^), lightweight (particle density between 500 and 2100 kg/m^3^), and ultra-lightweight (particle density less than 500 kg/m^3^) (Australia 2014) is primarily based on the density of the parent concrete. However, certain treatment methods can influence RA density by altering the amount of attached mortar or densifying the aggregate itself. Treatments like autogenous cleaning have yielded the most significant density reduction. During the treatment, the overall mass of RAs decreases due to the wearing of the mortar layer. The ball mill method results in the greatest density increase and can be effectively used if the parent concrete has relatively high density.

All TRA that have recorded density values fall within normal weight aggregate category. The studies mentioned in this review paper have provided data on bulk densities but the bulk density is always lower than particle density (Upadhyay and Raghubanshi 2020). The standard also suggests that aggregates with a water absorption greater than 2% may be acceptable given further investigation on the durability of the concrete manufactured (Xiao et al. 2022). Considering concrete performance in terms of mechanical and durability properties of structural application, methods for the removal of attached mortar, including ball mill crushing, concrete drum mixer crushing, pre-soaking in acetic acid, and strengthening methods such as accelerated carbonation using pure CO_2_, impregnation with PVA (air-dried), coating with F&S, F&C, NS, SAC, and BP achieve the best performance.

### Non-structural applications: sound insulation and concrete blocks

While TRA concrete may not always meet the stringent requirements for structural applications, it remains a valuable option for various non-structural uses where high porosity can be an advantage. For instance, the naturally higher porosity of TRAs makes them suitable for sound-insulating concrete in highway noise barriers (Han et al. 2023). This application benefits from the sound-absorptive properties of porous materials, and TRAs soaked in nano-CaCO₃ slurry or nano-SiO₂ solutions can further optimize porosity and water absorption. These treatments result in higher water absorption values, indicating increased porosity, which can enhance the material’s effectiveness in noise reduction. Additionally, using TRAs in the production of concrete blocks has shown promising results. Research by Poon et al. (2002) demonstrated that replacing NAs with RAs at levels of up to 50% had a negligible effect on compressive strength, making TRAs suitable for non-load-bearing applications like concrete blocks. At higher replacement levels, compressive strength may be slightly affected, but the ball mill method has proven particularly effective for improving compressive strength and water absorption, yielding TRAs that perform reliably in concrete mixes for block manufacturing. Despite these positive outcomes, it is worth noting that many studies have not thoroughly investigated how various treatment methods affect water absorption in TRA concrete mixes. Since water absorption is a critical factor for durability, especially in applications exposed to moisture, further research in this area would be valuable to better understand and optimize TRA performance in non-structural applications.

### Applications in transport infrastructure

Application of RAs in transport infrastructure has been successfully completed. A 9.3-km-long pavement section in the Interstate 10 (I-10) highway in Texas, USA, had been cast with 100% RA. Even after 10 years of service, excellent structural performance, without any distress has been recorded (Silva et al. 2019). The performance of each layer in a pavement is greatly influenced by the properties of materials utilized (Cardoso et al. 2016). Reis et al. (2021) states that RAs with higher densities can be used in low-volume roads as base and sub-base layers and their performance can be enhanced by using RAs containing cementitious materials. Coating with SAC with addition of BP could be a viable treatment option given it does not exceed the permissible sulphate content as Sulphur is known to contribute to the deterioration of concrete structures (Reis et al. 2021).

RAs can also be incorporated in manufacturing sustainable asphalt. Sanchez-Cotte et al. (2020) derived asphalt mixes with increased resilience modulus when 45% RAs substitution was employed. Álvarez et al. (2019) identified increased stability and satisfactory resistance to permanent deformation with up to 40% substitution. As per AS 2758.5:2020 (Australia 2020), a maximum LAAV of 30% is required for asphalt aggregates. Ball mill crushed and autogenously cleaned RAs have exhibited the highest reduction in LAAV rendering them most adaptable.

### Challenges in utilizing treatment methods in large-scale

Table 7 summarizes the recommended treatment methods for several practical construction applications based on physical, mechanical, and durability properties and the total duration of treatment methods.

**Table 7:** Recommended treatment methods for construction applications

Application	Recommended treatment methods	Time duration for treatment
Structural concrete elements	Removal methods: 1. Ball mill method 2. Concrete drum mixer crushing 3. Pre-soaking in acetic acid Strengthening methods: 1. Accelerated carbonation using pure CO_2_ 2. Impregnation with PVA (air-dried) 3. Coating with F&S, F&C, or NS 4. Coating with SAC and BP	Removal methods: 1. One hour 2. Three hours Strengthening methods: 1. Thirty hours 2. Eighty-four hours 3. Seventy-six hours 4. Seventy-two hours
Non-structural concrete: sound insulating elements	Strengthening methods: 1. Soaking in nano-CaCO₃ slurry or nano-SiO₂ solution	Seventy-three hours
Non-structural concrete: concrete blocks	Removal methods: 1. Ball mill method	One hour
Transport infrastructure: pavement	Strengthening methods: 1. Coating with SAC and BP	Seventy-two hours
Transport infrastructure: asphalt	Removal methods: 1. Ball mill method 2. Autogenous cleaning	Removal methods: 1. One hour 2. Ten hours

One of the primary barriers to large-scale adoption of RAs in concrete production is material variability, which affects consistency in the mechanical properties, durability, and overall performance. Unlike NAs, RAs originates from demolished concrete structures, making the quality highly dependent on source material, processing methods, and level of contamination. A parent concrete possessing a higher strength is more likely to generate RAs with a higher crushing value, making it more suitable for load-bearing applications. A lower-strength parent concrete produces RAs with weaker particles, higher porosity, and lower density, reducing its suitability for structural use (Pedro et al. 2017). In these scenarios, it can be beneficial to treat RAs to enhance their properties to achieve the desired performance and maintain consistency. Several contaminants in RAs such as plaster, gypsum, and sulfate compounds are known to cause delayed ettringite formation and thereby expansions in concrete (Tam and Tam 2007). Steel and other reinforcement material fragments can lead to corrosion issues if not properly removed (Kou and Poon 2009). Organic materials such as wood, plastic, and paper weaken bond strength and increase porosity (Kisku et al. 2017). These contaminants reduce workability, strength, and durability, requiring extensive material screening and processing. In addition, inconsistent RA quality makes it challenging to standardize concrete mix proportions, leading to batch-to-batch variation in large-scale concrete production (Silva et al. 2014). Advanced sorting techniques like X-ray fluorescence (XRF) and optical sorting, pre-washing and surface treatment techniques can be utilized to remove these contaminants. Additionally, processing costs remain a challenge for large-scale RA adoption, especially in regions where natural aggregates are readily available (Li et al. 2021a). Furthermore, lack of standardized RA treatment methods leads to inconsistency in engineering properties, making it difficult for concrete producers to ensure uniform quality (Silva et al. 2014). To address these issues, a performance-based acceptance criteria rather than fixed RA replacement limits can be specified.

## Effect of treatment methods on sustainability and cost considerations

The studies reviewed demonstrate that specific treatment methods can be effectively used in improving the performance of RA and potentially replace natural aggregates from concrete in future for a variety of applications in the construction industry. This can drastically reduce the adverse environmental impacts due to CDW and lower the consumption of natural resources (Patrisia et al. 2025). It also can directly contribute to promoting a circular economy by creating more efficient and sustainable products. Several studies have been conducted on life-cycle assessment (LCA) for RA products; however, these principally focus on mix design, the recycling process, and transportation factors, while treatment methods are rarely incorporated into the studies (Xing et al. 2022). The environmental consequences of physical methods significantly exceed those associated with chemical and biological methods, primarily due to the substantial generation of solid waste from physical techniques. This not only results in a reduced recovery rate of recycled materials but also contributes to air pollution (Feng et al. 2022). Thermo-mechanical processes for producing coarse RA consume 36 to 62 times more energy than standard recycling processes, particularly when microwave heating is not used (Xing et al. 2022). The LCA study by Pu et al. (2023) found that RAs processed using the standard two-stage crushing, and two-stage crushing combined with heat treatments increased the global warming potential (GWP) by 5.33% and 6.43%, respectively, compared to NC. In contrast, the accelerated carbonation treatment significantly reduced energy requirements, environmental impacts, and costs relative to natural aggregates. With 100% RA treated through carbonation, the GWP was 3.93% lower than that of NC. The source of CO_2_ for carbonation treatments also plays a pivotal role in the environmental impact. The utilization of flue gases aid in limiting greenhouse gas emissions to the atmosphere in comparison to commercially produced CO_2_. Treatments incorporating acidic solutions leave a trace of residual ions which requires an increased amount of water to clean them. This water must be treated separately in order to be discarded properly thus increasing energy consumption (Wang et al. 2017).

Treatment methods for RAs can significantly impact costs. The cost of the treatment can vary depending on the treatment procedure and additives. As per Table 7, treatment method and time can differ significantly. Energy-intensive processes such as oven-drying and sieving can lead to high energy costs. In terms of additives, the extensive use of cement leads to significantly higher costs for cement slurry impregnation treatment compared to alternative methods, rendering this approach economically less feasible (Feng et al. 2022). However, this could be minimized by partial substitution with waste pozzolans such as fly and slag. The costs associated with acid treatment are elevated due to the substantial volume and expense of raw materials, as well as the expenses incurred during the wastewater treatment process (Wang et al. 2017). Bio deposition is one of the cheapest technologies given that the raw materials are available from natural sources. Currently, the initial phase of research on bio deposition technology focuses on the cultivation of microorganisms, which incurs significant expenses. In reality, its application for enhancing resource recovery remains in the exploratory stage, necessitating extensive research before achieving an optimal process with limited costs (Feng et al. 2022). Although the NS spraying method features a straightforward application process, the cost of the nanomaterials remains relatively high. The combined treatment of mechanical abrasion and sodium silicate immersion, Ahmad et al. (2022), has been calculate to be more expensive than the single mechanical treatment; however, the benefits provide a significant resistance to sulphate attack and has been proven it to be a highly beneficial application in concrete elements in sulphate rich environments. Reduced energy consumption has been reported in the combined treatment of Ca^2+^-rich wastewater and carbonation by Fang et al. (2021), which highlights the adaptability of this methodology in practical application. The economic feasibility of using NS in TRA is low when applied via pre-soaking, as this method requires a large quantity of NS due to the high-water absorption of RA. Recently, a pre-spraying technique has been developed, applying NS to the surface of RA, and using significantly less NS than pre-soaking. However, given the high cost of NS, it is essential to evaluate the efficiency of the NS pre-spraying technique against other RA treatment methods before implementing it in practical applications (Li et al. 2021b).

## Summary, conclusions, and recommendations

The following conclusions can be drawn regarding the differing RA treatment techniques explored in past studies.

The ball mill method is the most effective method for mortar removal with regards to the physical and mechanical properties of aggregates and concrete. It also is the most suitable when manufacturing sustainable structural concrete, concrete blocks, and asphalt. Autogenously cleaned aggregates are also suitable for normal-weight concrete and asphalt, but their durability properties require further investigation.

Accelerated carbonation using pure CO_2_, PVA impregnation, coating with nano-CaCO_3_ with a dispersant, and F&S treatments are the most effective of the mortar strengthening techniques. PVA, acetic acid treatment and carbonation using pure CO_2_ are suitable in structural concrete applications. SAC and BP coatings are better in pavement construction. However, they also need more exploration with regards to their long-term properties.

The triple mixing method can be considered the best performing advanced mixing method in terms of mechanical properties. However, again the long-term performance needs to be investigated before incorporation into concrete infrastructure. As the mixing process is not a separate treatment for aggregates it could be much cheaper and energy-efficient than any other treatment method but lacks information to arrive at a definitive conclusion.

Combinations of two or more techniques have also proven to be highly effective in enhancing TRA properties both in the short term and the long term. It is an indication that if a single technique is not totally effective, it can be paired with other techniques to achieve an improved performance. However, this can create lengthy, and costly treatment procedures demanding high quantities of energy rendering them counterproductive.

Based on the review the following recommendations can be made for future studies of RA treatment methods to provide a holistic picture of their feasibility.

Only a handful of studies have ventured in to examine the durability properties of TRA concrete. Furthermore, they have done so primarily with regards to the resistance to chloride ion permeability, and occasionally, water absorption. Many additional properties such as creep, drying shrinkage, resistance to carbonation, resistance to sulphate attacks, resistance to freeze–thaw cycles are yet to be explored.

The experimental studies also have been confined to laboratory scale samples. In order to explore the feasibility of the practical application of TRA concrete, elements such as beams, columns, slabs, and their combinations should be explored. The crack patterns and failure modes need to be examined in more detail. In addition, to date there has been no research under extreme conditions such as exposure to fire, snow and sulphate rich environments.

To be used on a commercial scale, a comparison of costs and environmental impact should be conducted for all the treatment methods. Only the F&S treatment study has conducted a cost–benefit analysis and an environmental impact study. Life cycle assessment of costs and environmental impact in terms of total energy and carbon emissions need to be further explored.

In summary, this paper compares the different treatment techniques of strengthening recycled aggregate concrete. However, addressing the identified research gaps is crucial for developing materials suitable for the construction industry.

## Data Availability

All data generated or analyzed during this study are included in this published article and will be made available upon reasonable request.
